# Long-term labeling and imaging of synaptically connected neuronal networks in vivo using double-deletion-mutant rabies viruses

**DOI:** 10.1038/s41593-023-01545-8

**Published:** 2024-01-11

**Authors:** Lei Jin, Heather A. Sullivan, Mulangma Zhu, Thomas K. Lavin, Makoto Matsuyama, Xin Fu, Nicholas E. Lea, Ran Xu, YuanYuan Hou, Luca Rutigliani, Maxwell Pruner, Kelsey R. Babcock, Jacque Pak Kan Ip, Ming Hu, Tanya L. Daigle, Hongkui Zeng, Mriganka Sur, Guoping Feng, Ian R. Wickersham

**Affiliations:** 1 McGovern Institute for Brain Research, Massachusetts Institute of Technology, Cambridge, MA USA; 2 Department of Brain and Cognitive Sciences, Massachusetts Institute of Technology, Cambridge, MA USA; 3 Picower Institute for Learning and Memory, Massachusetts Institute of Technology, Cambridge, MA USA; 4 Allen Institute for Brain Science, Seattle, WA USA; 5 Stanley Center for Psychiatric Research, Broad Institute of MIT and Harvard, Cambridge, MA USA; 6 Present Address: Lingang Laboratory, Shanghai, China; 7 Present Address: School of Biomedical Sciences, The Chinese University of Hong Kong, Hong Kong, China; 8 Present Address: Department of Neuroscience, Baylor College of Medicine, Houston, TX USA

**Keywords:** Molecular neuroscience, Neural circuits

## Abstract

Rabies-virus-based monosynaptic tracing is a widely used technique for mapping neural circuitry, but its cytotoxicity has confined it primarily to anatomical applications. Here we present a second-generation system for labeling direct inputs to targeted neuronal populations with minimal toxicity, using double-deletion-mutant rabies viruses. Viral spread requires expression of both deleted viral genes in *trans* in postsynaptic source cells. Suppressing this expression with doxycycline following an initial period of viral replication reduces toxicity to postsynaptic cells. Longitudinal two-photon imaging in vivo indicated that over 90% of both presynaptic and source cells survived for the full 12-week course of imaging. Ex vivo whole-cell recordings at 5 weeks postinfection showed that the second-generation system perturbs input and source cells much less than the first-generation system. Finally, two-photon calcium imaging of labeled networks of visual cortex neurons showed that their visual response properties appeared normal for 10 weeks, the longest we followed them.

## Abstract (web summary)

Rabies-virus-based tracing is a widely used technique for mapping neural circuitry, but its cytotoxicity has limited its applications. Here Jin et al. present a second-generation system with minimal toxicity, using double-deletion-mutant rabies viruses.

## Main

Monosynaptic tracing, or the labeling of neurons in direct synaptic contact with a targeted neuronal population using a deletion-mutant neurotropic virus, has become an important technique in neuroscience since its introduction in 2007 (ref. ^1^) because it is the primary available means of brain-wide identification of directly connected neurons. Its core principles are as follows: (1) selective infection of a targeted group of neurons with a deletion-mutant neurotropic virus (which in almost all implementations to date is a rabies virus (RV) with the envelope glycoprotein gene G (National Center for Biotechnology Information (NCBI) gene symbol RABVgp4) deleted from its genome) and (2) complementation of the deletion in *trans* in the targeted starting cells so that the virus can fully replicate within them and spread, as wild-type RV does, retrogradely (in the central nervous system^2^) across those neurons’ input synapses to neurons directly presynaptic to them. This system has contributed to many discoveries about the synaptic organization of many systems in the mammalian nervous system.

With a few exceptions, monosynaptic tracing has primarily served simply as an anatomical tool for static identification of connected neurons, because the first-generation (∆G) rabies viral vectors that it is based on are swiftly cytotoxic^3–5^. This toxicity stems from the fact that the deletion of just the glycoprotein gene leaves the viral transcription and replication machinery intact. The virus still rapidly expresses its genes at high levels and replicates the viral core to high copy numbers^6^, perturbing endogenous gene expression^7,8^, inhibiting host cell protein synthesis^9^ and killing most infected neurons within approximately 2 weeks^3,4^.

Several efforts have been made to engineer less-toxic or nontoxic monosynaptic tracing systems. A first-generation system based on the CVS-N2c strain of RV appears to have lower toxicity than does the widely used SAD B19 strain^5^. More recently, it was reported that adding a destabilization domain to the C terminus of the viral nucleoprotein rendered the virus nontoxic, allowing monosynaptic tracing ‘with no adverse effects on neural physiology, circuit function and circuit-dependent computations’^10^. We have since shown that those results were probably obtained using reversion mutants that had lost the intended C-terminal addition^11^, although we also showed that the technique may be salvageable^11^, and the authors of the original study have persevered with their approach^12^. From our own laboratory, we have introduced second-generation (∆GL) rabies viral vectors, which have the viral polymerase gene L (for large protein; NCBI gene symbol RABVgp5) deleted along with G, and showed that they do not appear to perturb the structure or function of labeled neurons^4^. However, no one has previously shown that these vectors can spread between neurons. Although a baculovirus-based complementation system has recently been reported, it was not shown to work either in vivo or otherwise^13^.

Here we show that second-generation (∆GL) rabies viral vectors can be used for monosynaptic tracing of inputs to genetically defined populations of neurons, with the double deletion complemented by expression of both deleted viral genes in *trans* in the postsynaptic cells.

## Results

### Construction of a ∆GL-based monosynaptic tracing system

We planned for the second-generation monosynaptic tracing system to use the same pseudotyping strategy^1^ used in the first-generation one for targeting the initial RV infection to the postsynaptic starting cells (or source cells, defining these as RV-infected cells expressing the complementing gene(s) and therefore able to support viral replication). We began by making versions of our previously introduced second-generation (∆GL) rabies viral vectors^4^ packaged with the avian retroviral envelope protein EnvA, for selective infection of cells engineered to express EnvA’s receptor, referred to as TVA (tumor virus a)^14,15^.

Whereas the first-generation system typically relies on an adeno-associated virus (AAV) (referred to as a helper virus or helper AAV) for expression of the deleted glycoprotein gene in *trans* in the source cells^16–19^, the polymerase gene that is also deleted in the second-generation vectors is ~6.4 kb, too large^20–23^ for a straightforward extension of this approach. We also wanted expression of both the polymerase and the glycoprotein to be regulatable, because we anticipated that sustained expression and resulting viral replication could be toxic to source cells.

We, therefore, generated a knock-in mouse line, TRE-CB (short for TRE-tight-mCardinal-P2A-B19L), in which the genes for the RV polymerase (SAD B19 strain) and the red fluorophore mCardinal^24^ are under the control of the tetracycline response element^25^ in the TIGRE locus^26–28^. To interface with this polymerase allele, we developed a helper virus combination (now published for use with first-generation RV^19,29,30^) based on the Tet system. Specifically, one AAV, AAV1-syn-FLEX-splitTVA-EGFP-tTA is Cre-dependent^31^ (FLEX/DIO design^32^) and expresses TVA, the enhanced green fluorescent protein (EGFP)^33,34^ and the tetracycline transactivator (tTA), whereas a second AAV, AAV1-TREtight-mTagBFP2-B19G, expresses the RV glycoprotein and the blue fluorophore mTagBFP2 (ref. ^35^) under the control of the tetracycline response element. Our intent was that, when the helper viruses were used in the TRE-CB mouse with Cre expression in starting cells, the tTA expressed by the first AAV would drive expression both of G from the second AAV and of L from the knock-in allele. Note that the use of tTA makes this a Tet-Off system so that the genes are expressed by default, with optional administration of a tetracycline analog (for example, doxycycline) acting to suppress their expression.

∆GL viruses, by design, express genes at very low levels and are therefore intended to express recombinases, which allow many downstream applications even when expressed at low levels^4^. The viruses in this study therefore encoded either Cre (codon-optimized for mouse^36^) or Flpo (a codon-optimized version of the yeast recombinase Flp^37,38^). Another necessary component of the system was therefore a means of reporting Cre or Flpo activity, for which we used the Ai14 (ref. ^39^) and Ai65F (ref. ^28^) mouse lines, in which recombination causes tdTomato expression in cells expressing Cre or Flp, respectively.

Based on our extensive prior experiments titrating these helper viruses for use with first-generation RV to achieve high transsynaptic spread efficiency in Cre mice but low background without Cre^29,30^, for this second-generation system, we used the same helper virus concentrations and 7-d interval between AAV and RV injections as we have previously described for the first-generation one, only varying the survival time after RV injection.

### Labeling inputs to corticostriatal projection neurons

**Fig. 1:**
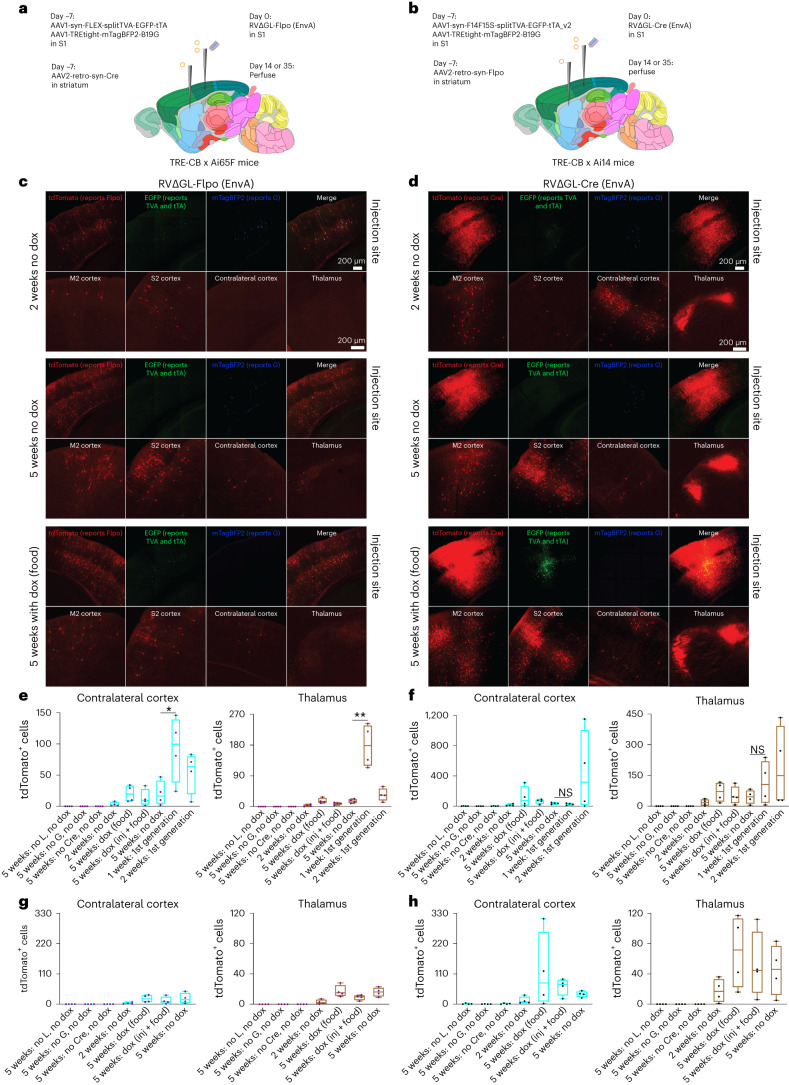
Second-generation monosynaptic tracing of inputs to corticostriatal neurons. **a**,**b**, Corticostriatal neurons were retrogradely targeted by an AAV2-retro expressing either Cre or Flpo injected in dorsolateral striatum, with a Cre- or Flp-dependent helper virus combination injected in S1. EnvA-enveloped ∆GL RV expressing either Flpo or Cre was injected in S1 7 d later. The mice were crosses of the TRE-CB line described here with tdTomato reporter lines to report the activity of RVs. **c**, Results using the Flpo-expressing RV. At 2 weeks, few cells were found in input regions, whereas by 5 weeks, substantial numbers of labeled cells were found in ipsilateral secondary motor and somatosensory cortices, in contralateral S1, and ipsilateral thalamus, with or without doxycycline (note that doxycycline suppresses expression of mTagBFP2 as well as the viral genes G and L). Scale bars: 200 µm, apply to all images. **d**, Results using the Cre-expressing RV. Images in **c** and **d** are representative of four independent experiments that yielded similar results. **e**,**f**, Counts of labeled cells in contralateral S1 and ipsilateral thalamus for RV∆GL-Flpo (**e**) and RV∆GL-Cre (**f**). Counts are the total number of cells found in a series of every sixth 50-µm section spanning each brain so that the true number of labeled neurons in the entirety of each brain would be approximately six times the number shown here. Although the first-generation Flp-dependent system at 2 weeks had on average labeled more neurons in input regions than any other condition (**f**), almost no source cells were found in the first-generation conditions at 2 weeks (Extended Data Fig. 3), suggesting that almost all source cells had died by that point. **g**,**h**, Same data as in **e** and **f** but with the first-generation counts omitted and with the ranges of the *y* axes of the Flpo and Cre versions set to the same value for each input region. Box plots depict the mean (center), first and third quartiles (lower and upper box limits) and minima and maxima (bottom and top whiskers). One-way ANOVAs were used for all data analysis in this figure (**e**,**f**). **indicates 0.001 ≤ *p* < 0.01; *indicates 0.01 ≤ *p* < 0.05.

We targeted corticostriatal cells in primary somatosensory cortex (S1) using rAAV2-retro (ref. ^40^) injected into dorsolateral striatum (Fig. 1), with helper AAVs and subsequently RV∆GL injected into S1. In one design (Fig. 1a), the rAAV2-retro expressed Cre, the helper AAVs were Cre-dependent as described above and the RV∆GL expressed Flpo. These injections were done in the Ai65F reporter line (Flp-dependent tdTomato) crossed to TRE-CB. In the other design (Fig. 1b), the rAAV2-retro expressed Flpo, the helper AAVs were Flpo-dependent (that is, the first helper virus contained orthogonal Flp recognition target (FRT) sites instead of lox sites) and the RV∆GL expressed Cre instead of Flpo. These injections were done in the Ai14 reporter line (Cre-dependent tdTomato) crossed to TRE-CB. Titers of AAV and RV vectors were matched across the two designs (Methods). To test the effect of using the Tet-Off mechanism to suppress G and L expression after an initial period of unrestricted viral replication, in some conditions we switched the mice to food containing doxycycline 2 weeks after RV injection (dox (food) conditions), whereas in others (the dox (injection + food) conditions) mice also received doxycycline by intraperitoneal injection for 3 d beginning at the same time as dox food was begun. Two to five weeks after RV injection, mice were perfused and the results were examined by confocal microscopy, with transsynaptic spread efficiency quantified by counting labeled neurons in contralateral cortex and ipsilateral thalamus.

**Extended Data Fig. 1:**
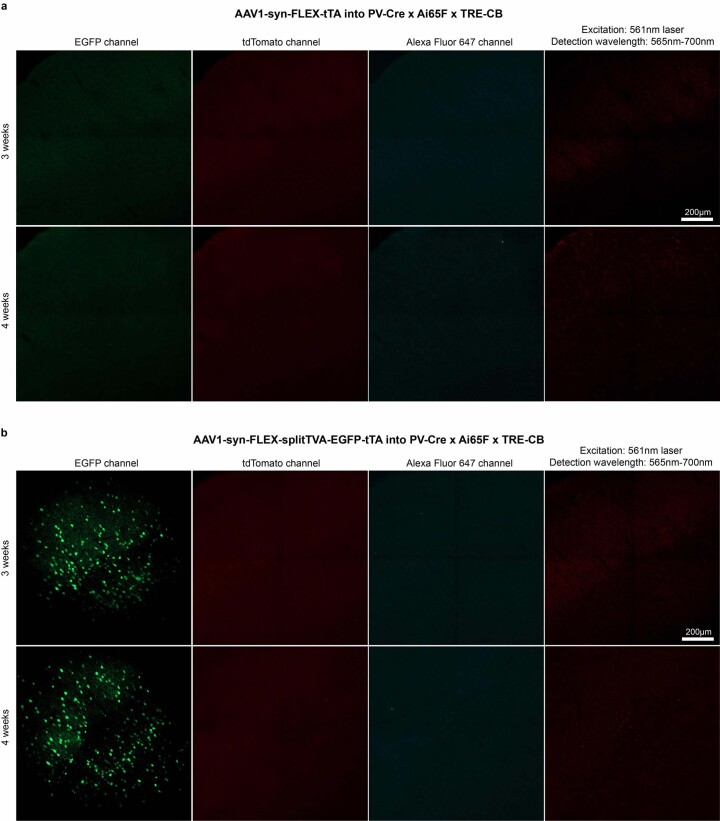
Intrinsic mCardinal fluorescence was not detected by confocal imaging, suggesting very low L expression in the conditions implemented. Representative confocal images of injection sites in PV-Cre x Ai65F x TRE-CB mice, showing lack of clear mCardinal fluorescence induced by either of the laser lines (561 nm or 640 nm) flanking the excitation maximum on the Zeiss LSM 900 confocal microscope. Because of the lack of fluorescence in the channel otherwise used for AlexaFluor 647, specifically, we were able to use that channel for immunostaining for PV and TH (Figs. 2,3 and Extended Data Figs. 5,6). **a**, Injection sites of a FLEX AAV expressing tTA without a fluorophore, and **b**, injection sites of the FLEX AAV expressing TVA, EGFP, and tTA, with survival times of 3 and 4 weeks in each case. Left: EGFP channel; center-left: tdTomato channel; center-right: AlexaFluor 647 channel; right: ‘best case’ for imaging mCardinal: excitation with 561 nm and collecting all emitted light between 565 nm and 700 nm. Scale bars: 200 µm, apply to all panels. Images in this figure are representative of four independent experiments that yielded similar results.

**Extended Data Fig. 2:**
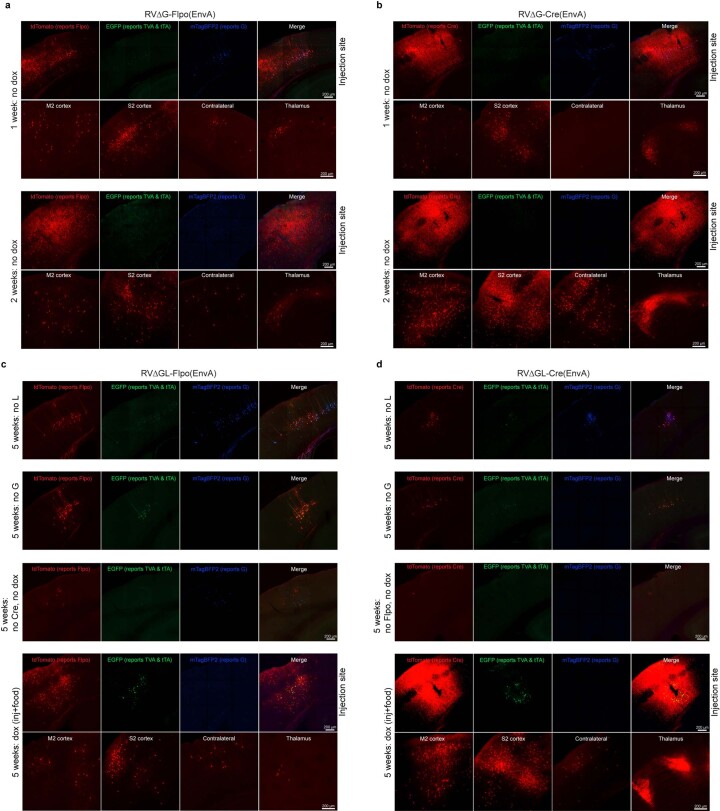
Representative images of control experiments for corticostriatal experiments. See Fig. 2 for explanation of experimental design and quantification. **a**,**b**, First-generation controls (7 d and 14 d survival times). **c**,**d**, Controls using second-generation viruses: no L, no G, and no Cre/Flpo controls (all without doxycycline), as well as the ‘dox (inj + food)’ condition for comparison. Scale bars: 200 µm, apply to all images. Images in this figure are representative of four independent experiments that yielded similar results.

With the Flpo-expressing RV∆GL, when all components were included, there were labeled neurons in the input regions at two different survival times (2 and 5 weeks after RV injection), with or without doxycycline (Fig. 1c,e,g; see Supplementary Table 1 for all cell counts and statistical comparisons). This spread of the ∆GL virus to input regions, increasing with time, was despite the fact that we were unable to see clear mCardinal signal, suggesting very low expression of L under the conditions implemented (Extended Data Fig. 1). First-generation control experiments using RV∆G-Flpo with the 7-d survival time that is typical for the first-generation system^29,41^ labeled significantly more cells in both input regions than any condition using the second-generation RV∆GL-Flpo (Fig. 1e; see Extended Data Fig. 2 for images from control conditions).

The second-generation system using the RV∆GL expressing Cre instead of Flpo resulted in label in input regions that was not significantly different than when using the corresponding first-generation one (Fig. 1d,f,h), although many of the comparisons were underpowered due to high variance and modest *n* (= 4 mice per condition throughout our study).

Control experiments in which either G or Cre/Flpo was omitted resulted in very few (at most four per series) labeled cells in input regions in either contralateral cortex or thalamus (Fig. 1e–h and Extended Data Fig. 2). This indicates that the apparent transsynaptic spread was not due to trivial confounding effects, such as direct retrograde infection by the TVA-expressing helper AAV followed by infection of the resulting TVA-expressing axons by the RV, or simply direct retrograde infection by residual RV coated with its native glycoprotein. For more discussion of such possible confounds, see ref. ^29^.

Crucially, control experiments in which L was omitted (by using double transgenics without the TRE-CB allele) resulted in no thalamic label with either virus, no contralateral cortical label with RV∆GL-Flpo and at most four labeled contralateral cortical neurons with RV∆GL-Cre (Fig. 1e–h and Extended Data Fig. 2). This indicates that complementation with both G and L is required for the ∆GL virus to replicate within the source cells and spread to presynaptic ones.

**Extended Data Fig. 3:**
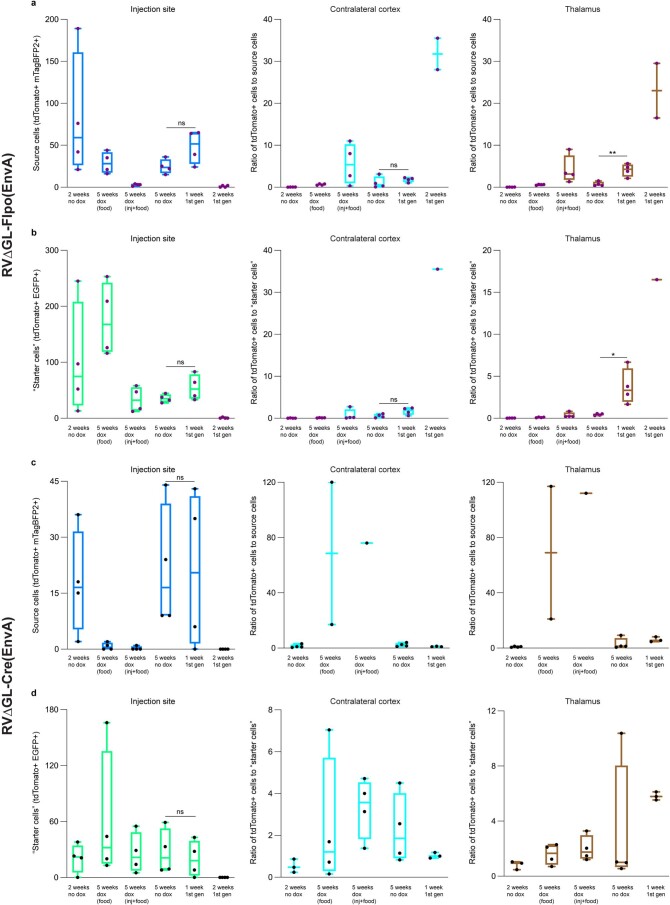
Counts of corticothalamic ‘source’ and ‘starter’ cells and ratios of contralateral and thalamic cells to source and starter cells. Counts are totals found in every sixth 50-µm section spanning each brain (n = 4 independent experiments for each condition). Box plots depict the mean (center), first and third quartiles (lower and upper box limits), and minima and maxima (bottom and top whiskers). One-way ANOVAs were used for all data analysis in this figure. **a**,**b**, Results using RV∆GL-Flpo(EnvA). **a**, Counts of source cells (left), defined as cells coexpressing tdTomato (marking activity of Flpo) and mTagBFP2 (marking expression of G), and ratios of these numbers of source cells to numbers of tdTomato+ cells in contralateral cortex (middle) and thalamus (right). Note the near-absence of source cells in the first-generation conditions at two weeks, suggesting that most had died by this time point. Note also that the low numbers of source cells in the second-generation conditions with doxycycline could reflect either cell death or simply the intended suppression of mTagBFP2 expression in these mice. For mice in which no source cells were found (either because they had died or because mTagBFP2 was suppressed by doxycycline), no ratio is included on the graphs. **b**, Counts and ratios of starting cells, defined here as cells coexpressing tdTomato (marking activity of Flpo) and EGFP (marking expression of TVA and tTA). **c**,**d**, Results using RV∆GL-Cre(EnvA). **c**, Counts and ratios of source cells, defined here as cells coexpressing tdTomato (marking activity of Cre) and mTagBFP2 (marking expression of G). **d**, Counts and ratios of starting cells, defined here as cells coexpressing tdTomato (marking activity of Cre) and EGFP (marking expression of TVA and tTA). ** indicates 0.001 ≤ *p* < 0.01; * indicates 0.01 ≤ *p* < 0.05.

Note that, although the first-generation RV∆G-Cre at 2 weeks postinjection had labeled more input neurons on average than any other condition (Fig. 1f), almost no source cells were found at this time point with either of the first-generation combinations (none in six of eight mice, and two cells each in two mice; Extended Data Fig. 3a,c and Supplementary Table 1), suggesting that the first-generation system had killed nearly all source cells by that point.

**Extended Data Fig. 4:**
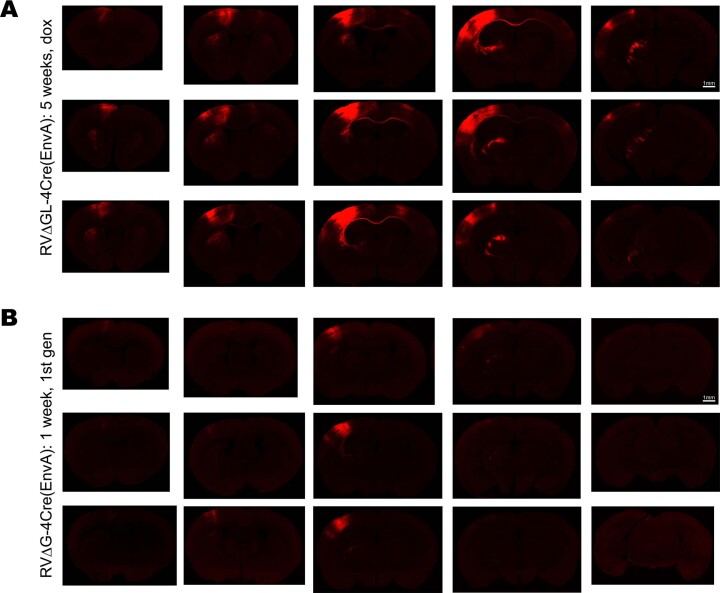
Series of whole-brain images of labeled inputs to corticostriatal neurons using first- and second-generation rabies viral vectors expressing Cre. Images are tiled confocal images of every sixth 50-µm coronal section spanning most of the rostrocaudal extents of the brains. **a**, Images from a mouse labeled using RV∆GL-Cre(EnvA) and associated AAVs (see Fig. 1b for experimental design), with a 5-week survival time and with doxycycline (administered in food only; see Methods). **b**, Images from a mouse labeled using RV∆G-Cre(EnvA) and associated AAVs, with a 1-week survival time. While the numbers of labeled neurons were not significantly different in either contralateral cortex or thalamus (see Fig. 1 and Supplementary Table 1), label is brighter in the ∆GL case, presumably because of greater tdTomato accumulation over the longer survival time. A similar difference can be seen when comparing the ∆G-Cre images in Extended Data Fig. 2b (top set of images) to the ∆GL-Cre ‘5 weeks with dox (food)’ images in Fig. 1d (bottom set of images).

Representative whole-brain image series of labeled inputs to corticostriatal neurons using the Cre-expressing first- and second-generation rabies viral vectors are shown in Extended Data Fig. 4.

### Labeling inputs in Cre mouse lines

**Fig. 2:**
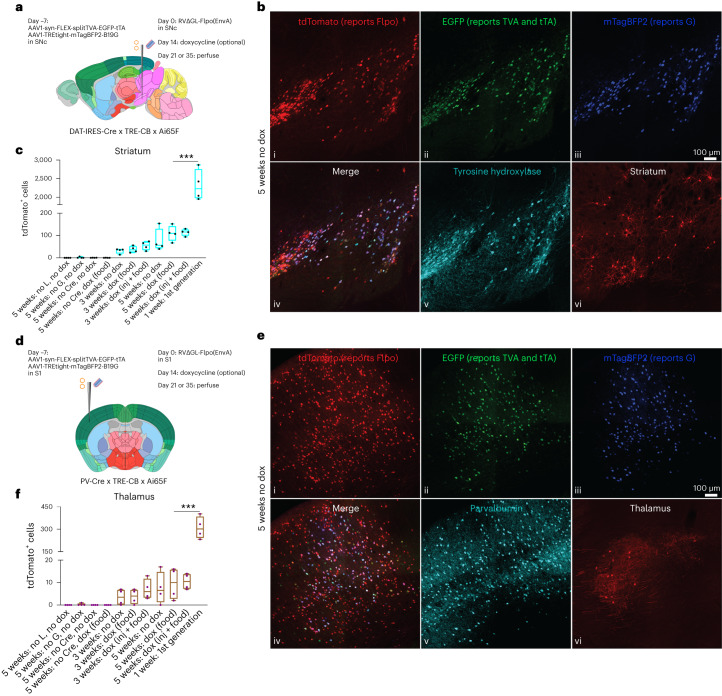
Second-generation monosynaptic tracing in Cre mice. **a**, Experiment design for labeling inputs to dopaminergic cells in SNc. The Cre-dependent helper virus combination was injected into SNc of triple transgenic mice (DAT-IRES-Cre x TRE-CB x Ai65F); EnvA-enveloped ∆GL RV expressing Flpo was injected 7 d later. **b**, Labeled cells 5 weeks after RV injection, without doxycycline. i–v, injection site in SNc, with many cells coexpressing tyrosine hydroxylase (indicating the dopaminergic cells), EGFP (expressed by the first helper virus), mTagBFP2 (expressed by the second helper virus) and tdTomato (reporting activity of the Flpo-expressing RV). Scale bar in iii: 100 µm, applies to all images; vi, medium spiny neurons in striatum labeled by the ∆GL RV. **c**, Counts of labeled striatal cells (total found in a series of every sixth 50-µm section spanning each brain) in all conditions tested. **d**, Experimental design for labeling inputs to parvalbumin-expressing cells in primary somatosensory cortex (S1) in PV-Cre x TRE-CB x Ai65F mice. **e**, Example of labeled cells 5 weeks after RV injection, without doxycycline. i–v, injection site in S1, with many cells coexpressing parvalbumin, reporters of both helper viruses and tdTomato reporting activity of the RV. In addition to these source cells, many putatively presynaptic cells expressing tdTomato are present. Scale bar in iii: 100 µm, applies to all images; vi, relay neurons in ipsilateral thalamus labeled by the ∆GL RV. **f**, Counts of labeled thalamic cells (total found in a series of every sixth 50-µm section spanning each brain) in all conditions tested. The first-generation system labeled many more cells than did this implementation of the second-generation system, potentially due to the low efficiency of recombination by Flpo in these reporter mice. Box plots depict mean, first and third quartiles and minima and maxima. One-way ANOVAs were used for all data analysis in this figure. ***indicates *p* < 0.001. Images in **b** and **e** are representative of four independent experiments that yielded similar results.

**Extended Data Fig. 5:**
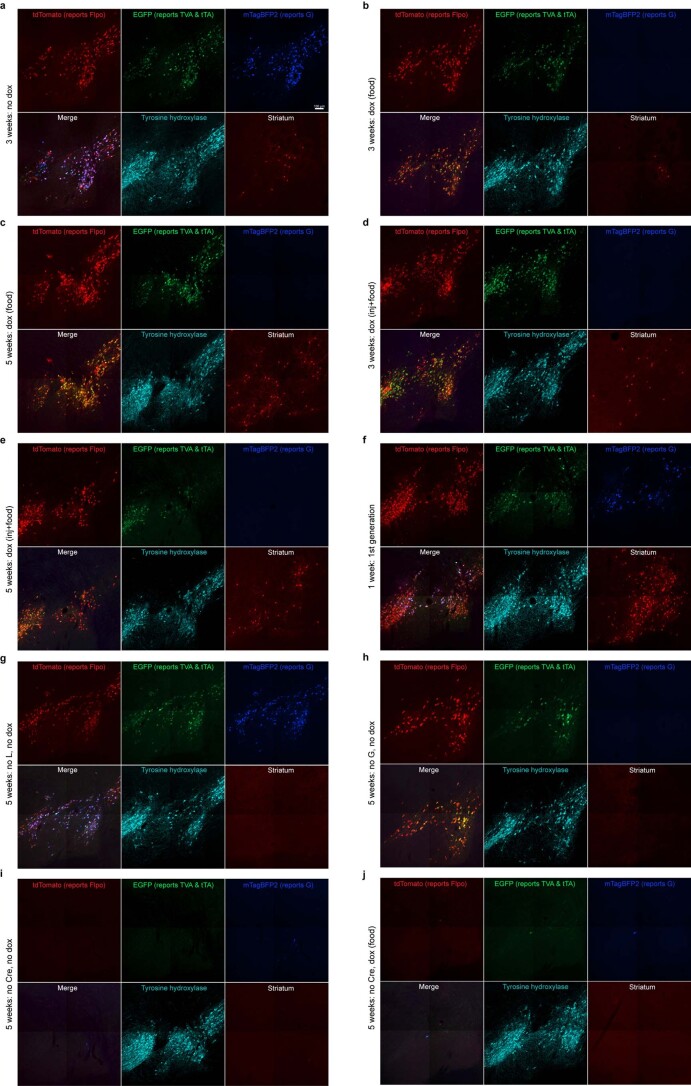
Representative images of second-generation monosynaptic tracing of inputs to dopaminergic midbrain neurons using RV∆GL-Flpo. See Fig. 1 for explanation of experimental design and for quantification. **a**, 3 weeks, no doxycycline, **b**, 3 weeks, with doxycycline in food, **c**, 5 weeks, with doxycycline in food, **d**, 3 weeks, with doxycycline in food and injected, **e**, 5 weeks, with doxycycline in food and injected. Control conditions are shown here: **f**, first-generation (∆G) system; **g**, no L; **h**, no G; **i**, no Cre, no doxycycline; **j**, no Cre, with doxycycline. Note that omitting L gave very similar results to omitting G. Images in this figure are representative of four independent experiments that yielded similar results.

**Extended Data Fig. 6:**
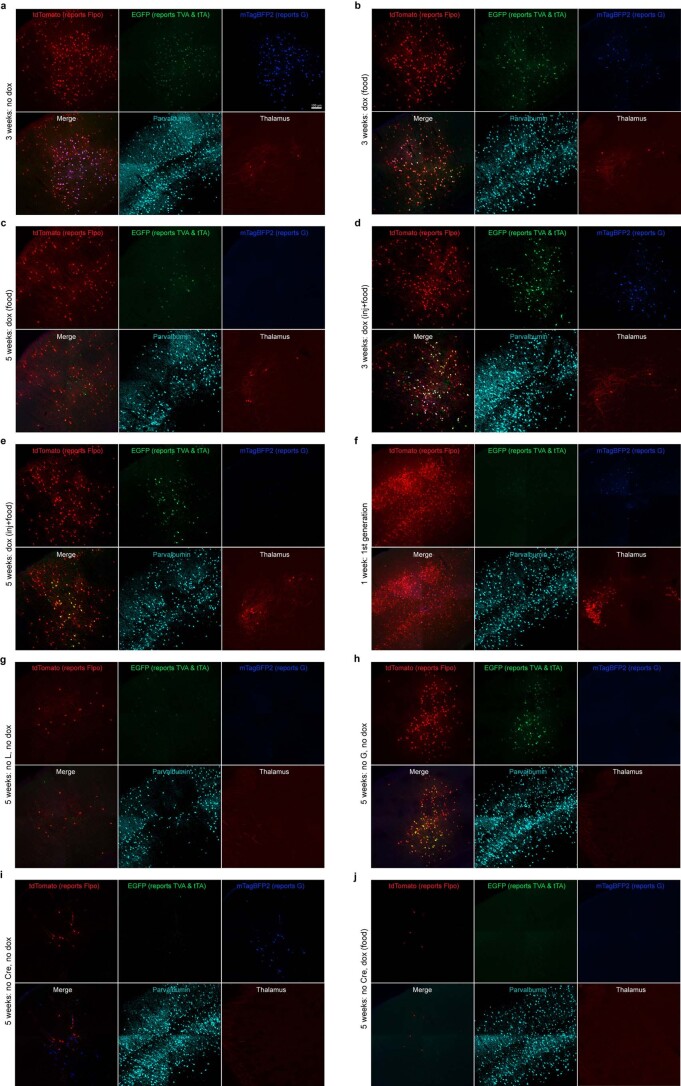
Representative images of second-generation monosynaptic tracing of inputs to parvalbumin-expressing cortical interneurons. See Fig. 1 for explanation of experimental design and for quantification. **a**, 3 weeks, no doxycycline; **b**, 3 weeks, with doxycycline in food (note that there is still blue fluorescence in this image, suggesting that, at one week after the mice were switched to dox food, the doxycycline has not completely suppressed G and presumably L expression); **c**, 5 weeks, with doxycycline in food; **d**, 3 weeks, with doxycycline in food and injected; **e**, 5 weeks, with doxycycline in food and injected. Control conditions are shown here: **f**) first-generation (∆G) system, **g**) no L, **h**) no G, **i**) no Cre, no doxycycline, **j**) no Cre, with doxycycline. Again, omitting L and omitting G gave very similar results. Images in this figure are representative of four independent experiments that yielded similar results.

**Extended Data Fig. 7:**
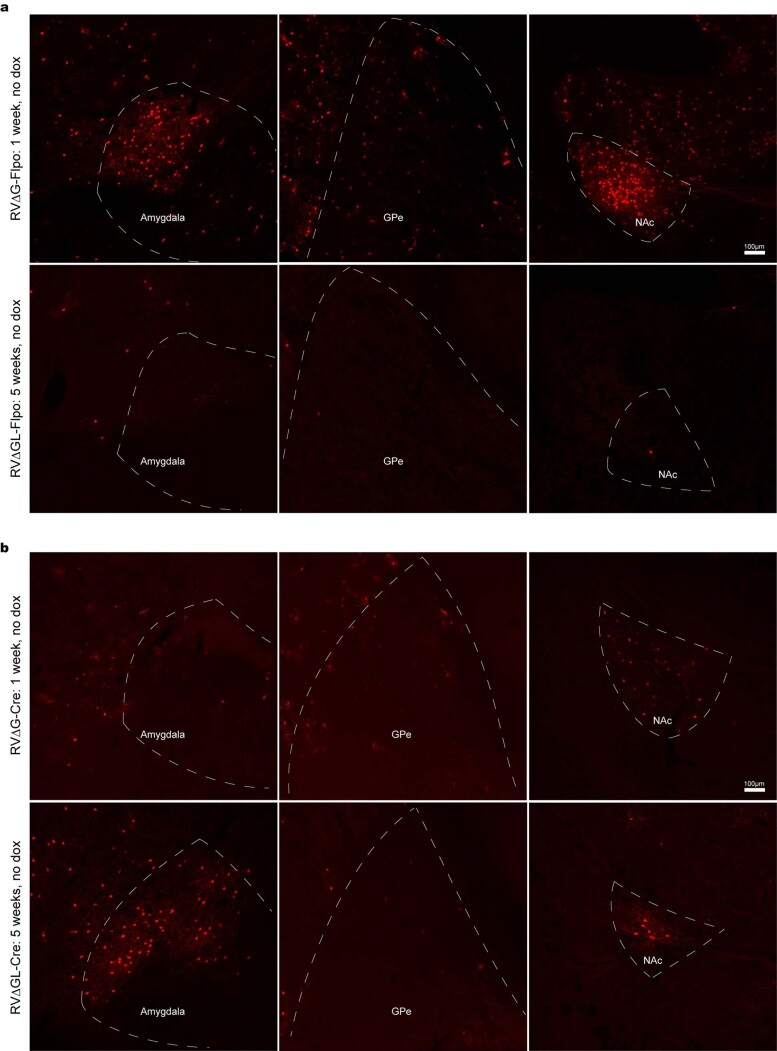
Examples of extrinsic inputs to dopaminergic cells labeled with first- and second-generation RV vectors expressing Flpo and Cre. **a**, Neurons labeled using ∆G and ∆GL rabies viruses expressing Flpo along with associated helper viruses (see Fig. 2a for experimental design). Top row: RV∆G-Flpo. Bottom row: RV∆GL-Flpo. **b**, Neurons labeled using ∆G and ∆GL rabies viruses expressing Cre along with associated helper viruses (see Fig. 3a for experimental design). Top row: RV∆G-Cre. Bottom row: RV∆GL-Cre. Scale bars: 100 µm, apply to all images.

**Extended Data Fig. 8:**
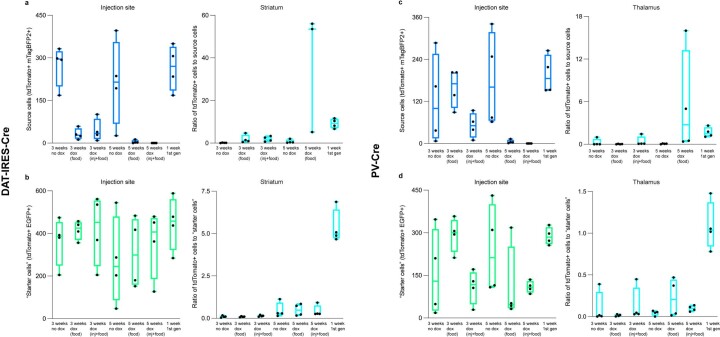
Counts of dopaminergic and parvabuminergic source/starter cells and ratios of striatal and thalamic cells to source/starter cells in Cre mice. Counts are totals found in every sixth 50-µm section spanning each brain (n = 4 independent experiments for each condition). Box plots depict the mean (center), first and third quartiles (lower and upper box limits), and minima and maxima (bottom and top whiskers). **a**,**b**, Results using RV∆GL-Flpo(EnvA) in DAT-IRES-Cre. **a**, Counts of source cells (left), defined as cells coexpressing tdTomato (marking activity of Flpo) and mTagBFP2 (marking expression of G), and ratios of these numbers of source cells to numbers of tdTomato+ cells in striatum (right). Note that the low numbers of source cells in the second-generation conditions with doxycycline could reflect either cell death or simply the intended suppression of mTagBFP2 expression in these mice. Note also that there is no graph for the ratio of labeled striatal cells to source cells for the ‘5 weeks dox (inj + food)’ because no source cells were found for that condition (that is, doxycycline appears to have fully suppressed mTagBFP2 expression). **b**, Counts and ratios of starter cells, defined here as cells coexpressing tdTomato (marking activity of Flpo) and EGFP (marking expression of TVA and tTA). **c**,**d**, Results using RV∆GL-Flpo(EnvA) in PV-Cre. **c**, Counts and ratios of source cells, defined here as cells coexpressing tdTomato (marking activity of Flpo) and mTagBFP2 (marking expression of G). Note that again there is no graph for the ratio of labeled contralateral cells to source cells for the ‘5 weeks dox (inj + food)’ because no source cells were found for that condition. **d**, Counts and ratios of starter cells, defined here as cells coexpressing tdTomato (marking activity of Flpo) and EGFP (marking expression of TVA and tTA).

We tested the system in the following two widely used Cre mouse lines: DAT-IRES-Cre^42^ and PV-Cre^43^, crossed to TRE-CB and the Flp reporter line Ai65F (Fig. 2 and Extended Data Figs. 5–8). In DAT-IRES-Cre, in which Cre is expressed in dopaminergic cells in the midbrain, we injected the viruses in substantia nigra pars compacta (SNc; Fig. 2a). In PV-Cre, in which Cre is expressed in parvalbumin-expressing cells in cortex and elsewhere, we injected primary somatosensory cortex (S1; Fig. 2d).

In both mouse lines, with or without doxycycline, we found spread of RV to regions known to provide input to the respective populations. In DAT-IRES-Cre, we found labeled neurons in striatum (Fig. 2b) and cortex. In PV-Cre, we found labeled neurons in thalamus (Fig. 2e) and other cortical areas, among other locations. In both mouse lines, just as with the corticostriatal experiments described above, there were more neurons at a later time point (5 weeks following RV injection) than at an earlier one (3 weeks; see Supplementary Tables 2 and 3 for all cell counts and statistical comparisons). Because pilot studies had suggested that survival times of 1 or 2 weeks did not result in substantial spread and that survival times of 7 weeks and higher did not result in much more spread than at 5 weeks, we only compared 3- and 5-week survival times for this set of quantitative experiments.

Administration of doxycycline beginning 2 weeks after RV injection did not result in significantly different amounts of label in input regions in either mouse line at either survival time, although these comparisons were again underpowered. This was contrary to our expectation that this intervention, which was designed to reduce toxicity to source cells by shutting off viral replication after an initial period, could also reduce the efficiency of transsynaptic spread.

Comparison to the first-generation system in the same Cre lines, however, showed that this implementation of the second-generation one appeared to be far less efficient—matched experiments using a first-generation virus, RV∆G-Flpo (and in mice without the TRE-CB allele), with a 7-d survival time, labeled 1.3–1.5 orders of magnitude (~21× in DAT-IRES-Cre and ~33× in PV-Cre) more neurons in input regions (Fig. 2c,f).

The results of the control experiments with both mouse lines were as expected—omission of any of G, L or Cre resulted in almost no labeled cells in input regions (Fig. 2 and Extended Data Figs. 5 and 6).

### Mapping inputs to dopaminergic neurons in a Flpo mouse line

**Fig. 3:**
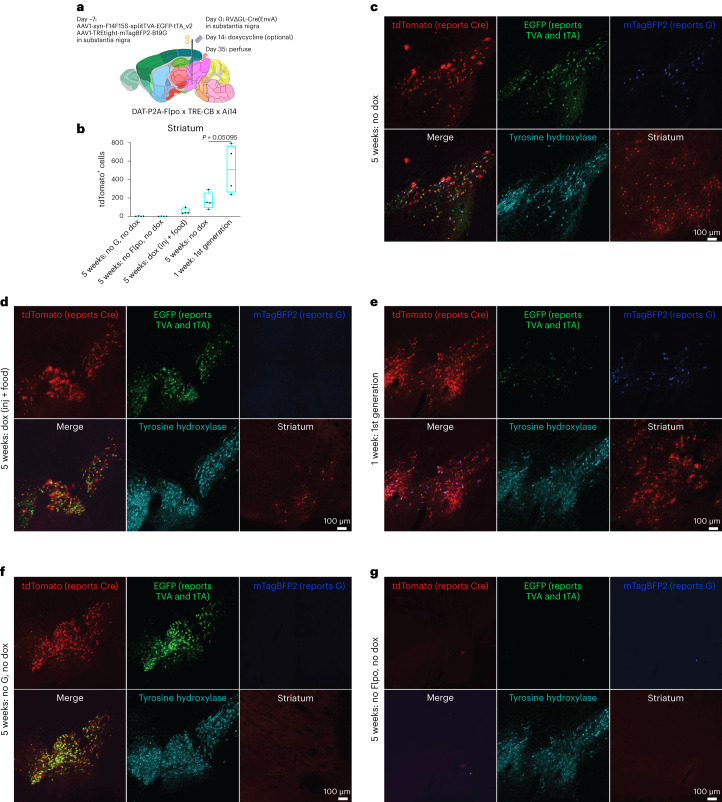
Second-generation monosynaptic tracing in DAT-P2A-Flpo mice. **a**, The design of the experiment was similar to that shown in Fig. 2a but using the Flp-dependent helper virus combination, and Cre-expressing RV, in DAT-P2A-Flpo x TRE-CB x Ai14 mice. **b**, Counts of labeled striatal neurons (total found in a series of every sixth 50-µm section spanning each brain) in all conditions tested (*n* = 4 independent experiments for each condition). Box plots depict mean, first and third quartiles and minima and maxima. Comparison of 5-week no dox and first-generation conditions: one-way ANOVA, *P* = 0.05095. **c**, Labeled cells 5 weeks after RV injection, without doxycycline. Many cells at the injection site in SNc were found to coexpress tyrosine hydroxylase and the fluorophores expressed by the helper viruses, as well as tdTomato, reporting activity of the Cre-expressing RV. Medium spiny neurons in striatum were labeled by the ∆GL RV. **d**, Example of a doxycycline (injection + food) mouse. The numbers of striatal input neurons were not significantly lower in this condition than without doxycycline, although comparisons were underpowered. **e**, Example label from the first-generation system. The numbers of striatal input neurons were not significantly higher than with the second-generation system, although comparisons were again underpowered. See also Extended Data Fig. 10 for whole-hemisphere image series for RV∆G-Cre and RV∆GL-Cre. **f**,**g**, Example of control conditions in which either G (**f**) or Flpo (**g**) was omitted. Scale bars: 100 µm, apply to all images. Images from **c** to **g** are representative of four independent experiments that yielded similar results.

**Extended Data Fig. 9:**
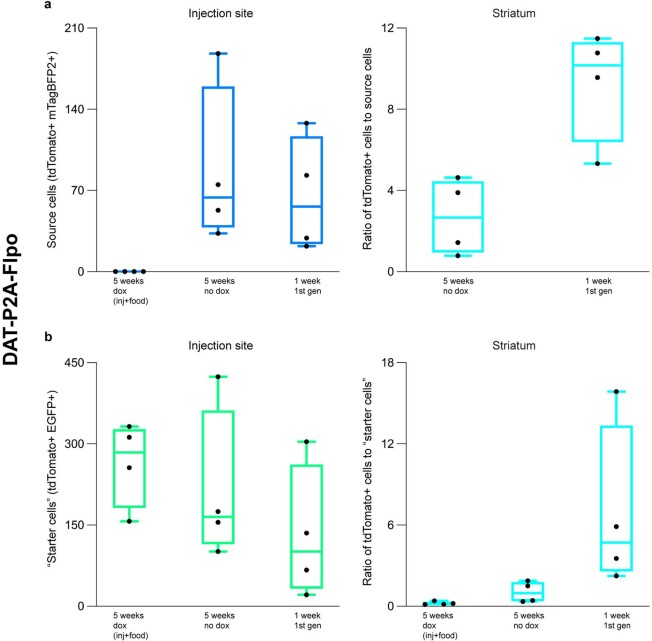
Counts of dopaminergic and parvabuminergic source/starter cells and ratios of striatal cells to source/starter cells using RV∆GL-Cre(EnvA) in DAT-P2A-Flpo mice. Counts are totals found in every sixth 50-µm section spanning each brain (n = 4 independent experiments for each condition). Box plots depict the mean (center), first and third quartiles (lower and upper box limits), and minima and maxima (bottom and top whiskers). **a**, Counts of source cells (left), defined as cells coexpressing tdTomato (marking activity of Cre) and mTagBFP2 (marking expression of G), and ratios of these numbers of source cells to numbers of tdTomato+ cells in striatum (right). Note that the low numbers of source cells in the second-generation condition with doxycycline could reflect either cell death or simply the intended suppression of mTagBFP2 expression in these mice. **b**, Counts and ratios of starter cells, defined here as cells coexpressing tdTomato (marking activity of Cre) and EGFP (marking expression of TVA and tTA).

**Extended Data Fig. 10:**
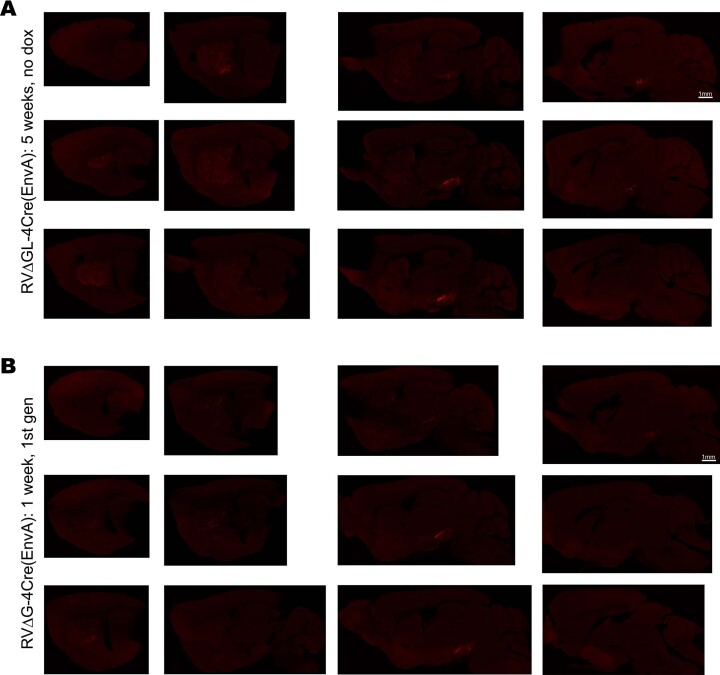
Whole-hemisphere image series of labeled inputs to dopaminergic neurons using first- and second-generation rabies viral vectors expressing Cre. Images are tiled confocal images of every sixth 50-µm parasagittal section spanning the injected hemispheres. **a**, Images from a mouse labeled using RV∆GL-Cre(EnvA) and associated AAVs (see Fig. 3a for experimental design), with a 5-week survival time and with doxycycline (administered in food; see Methods). **b**, Images from a mouse labeled using RV∆G-Cre(EnvA) and associated AAVs, with a 1-week survival time.

Because the Flp-dependent version of the system using RV∆GL-Cre resulted in more labeled input neurons than the Cre-dependent one using RV∆GL-Flpo in the corticostriatal experiments (Fig. 1), we tested the Flp-dependent system in DAT-P2A-Flpo mice (crossed to TRE-CB and Ai14), again injecting helper AAVs and RVs in SNc (Fig. 3a). We again found the expected patterns of label at the injection site and in the striatum and other input regions (Fig. 3b–d), and controls in which either G or Flpo were omitted resulted in very few (0–5) labeled cells in striatum (Fig. 3b,f,g). Without doxycycline, the numbers of labeled striatal cells were not significantly higher with the Flp-dependent system in this mouse line than with the corresponding Cre-dependent condition in DAT-IRES-Cre mice (RV∆GL-Cre in DAT-Flpo: mean 167 striatal cells; RV∆GL-Flpo in DAT-IRES-Cre: mean 76.75 striatal cells; *P* = 0.134, *n* = 4 mice per condition; see Supplementary Table 4 for counts and statistical comparisons). With doxycycline, the numbers were lower than in the corresponding Cre-dependent condition, a difference that was significant. The first-generation system did not label significantly more striatal cells than did the second-generation system (Fig. 3b,e; RV∆GL-Cre 5 weeks no dox: mean 167 striatal cells; RV∆G-Cre 1 week: mean 511.5 striatal cells; *P* = 0.051, *n* = 4 mice per condition). See also Extended Data Fig. 7 for images of label in several input regions (amygdala, external globus pallidus and nucleus accumbens) for both ∆G and ∆GL viruses expressing Flpo and Cre, Extended Data Fig. 9 for source/starter cell counts and ratios and Extended Data Fig. 10 for whole-brain image series for RV∆G-Cre and RV∆GL-Cre.

### Longitudinal two-photon imaging of labeled networks

We conducted several experiments to assay the survival and physiological status of neurons labeled with the second-generation monosynaptic tracing system. In previous work, we used longitudinal two-photon microscopy to show that neurons labeled with a second-generation (∆GL) RV stayed alive for as long as we imaged them and also that it did not appear to perturb their visual response properties^4^. Because we had only used the Cre-expressing versions of ∆G and ∆GL viruses for the longitudinal two-photon imaging work from our previous publications^4,11^, we first conducted a similar set of experiments using the Flpo-expressing RV∆GL and confirmed that it was similarly nontoxic (Supplementary Fig. 1). These findings suggested that second-generation monosynaptic tracing should be quite nonperturbative to labeled presynaptic neurons (which are only infected with the ∆GL viruses and not the helper viruses), except perhaps to the extent that toxicity to the postsynaptic source cells might perturb cells presynaptic to them by trophic or other network effects.

**Fig. 4:**
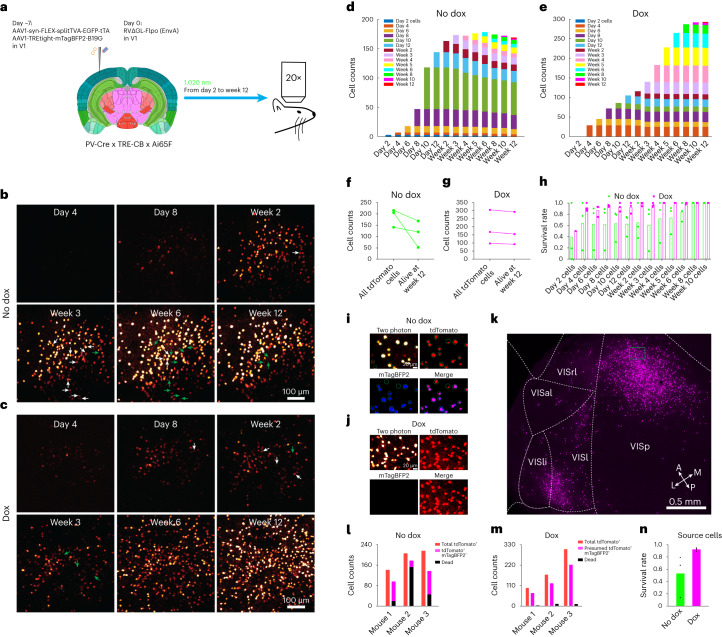
Longitudinal structural two-photon imaging in vivo. **a**, Experiment design. **b**,**c**, Two-photon images of tdTomato-labeled neurons in the V1 injection site at different time points. Arrows indicate example cells that are alive up to one time point (white arrows) but that are missing subsequently (green arrows). In this example, 46 of 215 cells were lost in the ‘no-dox’ mouse, whereas 12 of 305 cells were lost in the ‘dox’ mouse. Scale bar: 100 μm. **d**, Cell counts from all time points in a ‘no-dox’ mouse. Bar height for each color indicates the number of cells that were first visible at the corresponding time point that are still visible at the time point shown on the *x* axis. **e**, Cell counts from all time points in a ‘Dox’ mouse. **f**, Total number of imaged cells and number of cells present at the last imaging time point for each ‘no-dox’ mouse. **g**, Total number of imaged cells and number of cells present at the last imaging time point for each ‘dox’ mouse. **h**, Survival rates of cells that appeared at each time point for each mouse in both ‘dox’ and ‘no-dox’ groups. **i**, Aligned 12-week two-photon and postmortem confocal images from a ‘no-dox’ mouse. Scale bar: 20 µm. **j**, Aligned 12-week two-photon and confocal images from a ‘Dox’ mouse. Scale bar: 20 µm. **k**, Large-scale confocal image of labeled neurons in multiple visual cortical areas in the same ‘Dox’ mouse. Green rectangle indicates the FOV shown in **j**. Images from **b**, **c**, **i**–**k** are representative of three independent experiments that yielded similar results. **l**, Counts for each ‘no-dox’ mouse of the total number of imaged tdTomato^+^ cells (red), the number of cells that disappeared (black) and the number of cells found by postmortem confocal imaging to have expressed both tdTomato and mTagBFP2, considered surviving source cells (magenta). **m**, Counts for each ‘dox’ mouse of the total number of imaged tdTomato^+^ cells (red), the number of cells that disappeared (black) and the assumed number of total source cells (magenta). **n**, Estimated source cell survival rates for ‘dox’ and ‘no-dox’ groups.

We then conducted an imaging experiment to assay how toxic the full second-generation monosynaptic tracing system was to neurons, including the source cells, in which G and L were provided (Fig. 4). Similar to the experiments presented in Fig. 2d–f, we injected the Cre-dependent helper virus combination into visual cortex of PV-Cre x TRE-CB x Ai65F mice, followed by RV∆GL-Flpo(EnvA) 7 d later. For this set of experiments, we additionally implanted a headmount and optical window (Methods), and we imaged fields of view at or near the injection sites on a two-photon microscope repeatedly over 14 consecutive sessions beginning at 2 d postinjection and ending at 12 weeks (Fig. 4a). Just as for the previous experiments, half of the mice were given doxycycline beginning at 2 weeks postinjection.

We found that, with or without doxycycline, the majority of tdTomato-labeled cells at the injection sites survived for the full 12 weeks of imaging in most mice. For the mice that did not receive doxycycline and that therefore had G and L expressing throughout the 12-week experiment, 63.4% of labeled cells survived through the final imaging session. For the mice that did receive doxycycline, however, 94.4% of labeled neurons survived (Fig. 4b–h and Supplementary Fig. 2; see also Supplementary Table 6 for cell counts). Again, despite doxycycline administration, transsynaptic spread to other cortical areas was still widespread in these mice (Fig. 4k).

Although the overall cell survival rate was high, we anticipated that it would be lower for source neurons, in which G and L are provided and viral replication is therefore restored. To determine the survival rate of source cells, the ideal approach would have been to image both tdTomato (reporting activity of the Flpo expressed by the RV) and mTagBFP2 (coexpressed with G by the second helper virus)—neurons that expressed both fluorophores would be both infected by the RV∆GL and expressing G (and presumably L, because both depend on tTA expression) and therefore competent hosts for the RV to replicate within and spread to cells presynaptic to them. However, because the two-photon microscope to which we had access was unfortunately incapable of producing short-wavelength light at sufficient power to allow imaging of mTagBFP2 in vivo, we had to rely on indirect measures to estimate the source cells’ survival rates. Note that EGFP, which was coexpressed with TVA, is not a good indication of whether a cell is a source cell, because, although some of the cells expressing TVA would have been directly infected by the EnvA-enveloped RV, only a subset of such cells also express G (as reported by mTagBFP2; Supplementary Table 3) and L and are therefore able to support replication and spread of the RV. In any case, at the helper virus concentrations used^29,30^, the intrinsic EGFP signal is quite weak and needs to be amplified with immunostaining to be clearly discernible.

Following longitudinal two-photon imaging of tdTomato-labeled cells over the full 12 weeks, therefore, we perfused the mice, sectioned the brains in a plane approximately parallel to the imaging plane and then imaged the same regions using confocal microscopy. This allowed us to image both tdTomato and mTagBFP2 (for animals that had not received doxycycline) and then align the confocal images with the two-photon ones from the 12-week time point to identify the surviving double-labeled source cells (Fig. 4i,j). For those animals that did receive doxycycline, the blue channel contained little signal, as expected (Fig. 4j). Our second goal for the confocal imaging, however, was to confirm that transsynaptic spread in these mice used for two-photon imaging had occurred, which it had (Fig. 4k). For both dox and no-dox conditions, we made the assumption that all cells that had died were source cells, based on our finding that cells infected solely with RV∆GL-Flpo without complementation almost always survive (Supplementary Fig. 1; see also ref. ^4^ for extensive characterization of the nontoxicity of ∆GL RV).

In the mice that did not receive doxycycline, in which the surviving source cells (that is, those expressing red and blue fluorophores) were visible by confocal microscopy (Fig. 4i), we estimated the source cell survival rate in each mouse as the ratio of the number of cells in the field of view expressing both red and blue fluorophores divided by the sum of that number plus the number of cells that died in that field of view (Fig. 4l,n; see Supplementary Table 6 for cell counts and calculations). For these no-dox mice, we obtained a survival rate of 53.2% of source cells.

In the mice that did receive doxycycline, in which surviving source cells were not identifiable as such by postmortem confocal microscopy because they no longer expressed mTagBFP2 (Fig. 4j), we made the assumption that source cells made up the same percentage of total tdTomato-labeled neurons in the fields of view in the dox mice as in the no-dox ones because the experimental conditions were identical apart from the switch to doxycycline 2 weeks after RV injection. This gave us an estimated number of total source cells for each mouse. The difference between that and the (small) number of neurons that had disappeared over the course of the two-photon imaging gave us an estimated number of surviving source cells. The ratio of the estimated number of surviving source cells to the estimated total number of source cells was our estimate of the source cell survival rate (Fig. 4m,n; see Supplementary Table 6 for cell counts and calculations). For these doxycycline mice, we obtained an estimated survival rate of 92.3% of source cells.

### Ex vivo whole-cell recordings from input and source neurons

**Fig. 5:**
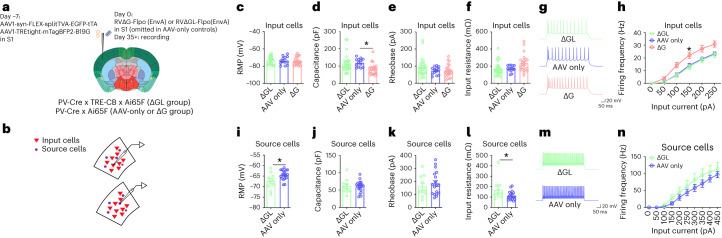
Membrane properties of cells labeled by first- and second-generation monosynaptic tracing. **a**,**b**, Experimental design. Beginning 35 d after RV injection, labeled neurons (input cells, defined as pyramidal neurons expressing tdTomato alone, or source cells, defined as neurons expressing both tdTomato and mTagBFP2) were targeted for ex vivo whole-cell recording. For AAV-only mice, so-called source cells denote neurons expressing mTagBFP2 and so-called input cells denote nearby unlabeled pyramidal neurons. **c**–**h**, Membrane properties of input cells (defined above). **c**, RMP; **d**, capacitance; **e**, rheobase and **f**, input resistance (Rm) of input cells. ΔGL, 26 cells from four mice; control, 13 cells from three mice; ΔG, 22 cells from three mice; RMP, one-way ANOVA, *F*(2, 58) = 0.35, *P* = 0.71. Capacitance, one-way ANOVA, *F*(2, 58) = 4.924, *p* = 0.011, Dunnett’s multiple comparisons test, ΔGL versus control, *P* = 0.93, ΔG versus control, *p = 0.023. Rheobase, one-way ANOVA, *F*(2, 58) = 1.81, *p* = 0.17. Rm, one-way ANOVA, *F*(2, 58) = 4.13, *P* = 0.021, Dunnett’s multiple comparisons test, ΔGL versus control, *p* > 0.99, ΔG versus control, *p* = 0.060. **g**, Representative traces of input cells’ firing in response to 250 pA current injection. **h**, Input cells’ firing frequency in response to sequential current step injection. Two-way ANOVA, *F*(2, 52) = 5.61, *P* = 0.0062. Dunnett’s multiple comparisons test, ΔGL versus AAV control, *p* = 0.94, ΔG versus AAV control, *p = 0.031. **i**–**n**, Membrane properties of source cells (defined above) in the ΔGL and control groups. Note that no surviving source cells could be found in the ∆G group at this survival time. **i**, RMP; **j**, capacitance; **k**, rheobase; **l**, Rm of source cells. ΔGL, 10 cells from four mice; control, 19 cells from three mice; RMP, unpaired *t* tests (two-tailed), *p = 0.016. Capacitance, unpaired *t* tests (two-tailed), *p* = 0.95. Rheobase, unpaired *t* tests (two-tailed), *p* = 0.47. Rm, unpaired *t* tests (two-tailed), *p = 0.040. **m**, Representative traces of source cell firing in response to 450 pA current injection. **n**, Source cells’ firing frequency in response to sequential current step injection. Two-way ANOVA, *F*(1, 27) = 1.42, *p* = 0.24. Error bars: mean ± s.e.m.

To further assay the degree to which the second-generation monosynaptic tracing system perturbs input and source neurons, we performed whole-cell recordings in acute brain slices from mice in which inputs to parvalbumin-expressing cortical neurons had been labeled (Fig. 5). As with the anatomical experiments (Fig. 2), we injected Cre-dependent helper viruses in primary sensory cortex of PV-Cre x TRE-CB x Ai65F mice, followed by either RV∆G-Cre(EnvA) or RV∆GL-Cre(EnvA; or no RV for AAV-only controls) 7 d later (Fig. 5a). Beginning 35 days after RV injection, we made slices from the injected brains and conducted whole-cell recordings from labeled input cells and source cells (Fig. 5b). For the AAV-only control group, the so-called input cells were randomly selected pyramidal neurons near the AAV-labeled neurons (Methods).

In brains labeled by the second-generation system, we found that the input cells did not differ from those of unlabeled neurons in the AAV-only control mice in all five measured membrane properties (Fig. 5c–h, resting membrane potential (RMP), capacitance, rheobase, input resistance and firing frequency), and that source cells differed from so-called source cells in the AAV-only control mice (that is, cells labeled by the helper AAVs) in two of the five measured membrane properties (RMP and input resistance, Fig. 5i–n).

By contrast, in brains labeled by the first-generation system, those input cells that remained at the time of recording (5 weeks after infection) differed from unlabeled control neurons in three of five measured membrane properties (capacitance, input resistance and firing frequency; Fig. 5c–h; cf. ref. ^4^). Furthermore, no remaining source cells could be found at all in the first-generation-labeled brains at this long survival time.

### Two-photon imaging of labeled neurons’ visual responses

**Fig. 6:**
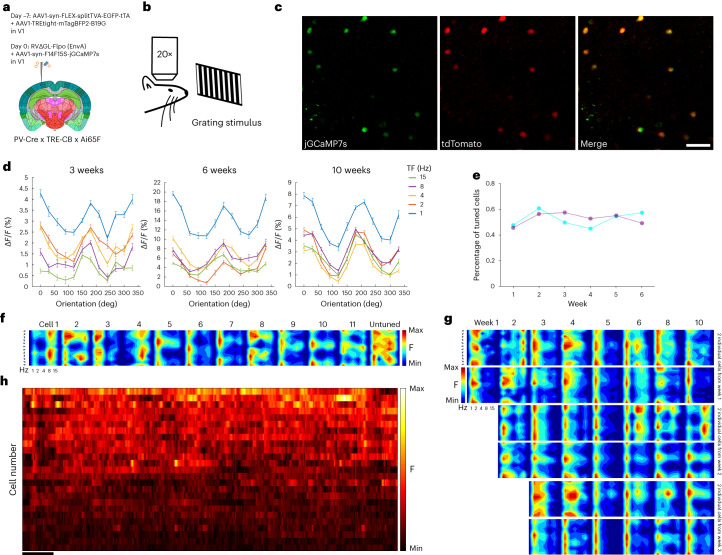
Functional two-photon imaging of transsynaptically labeled neurons’ visual response properties over 10 weeks. **a**, The experimental design was similar to that shown in Fig. 4 but with a Flp-dependent jGCaMP7s AAV injected along with the RV. **b**, Following the virus injections, the injection site was imaged on a two-photon microscope while the awake mice were presented with drifting grating stimuli of different orientations and TFs, repeatedly for 10 weeks after RV injection. **c**, Representative two-photon field of view of neurons expressing jGCaMP7s (green channel) and tdTomato (red channel). Scale bar: 50 µm. Images from **c** are representative of two independent experiments that yielded similar results. **d**, Tuning curves of a jGCaMP7s-expressing neuron obtained with drifting gratings presented at 12 directions of motion and five TFs, repeated ten times (mean Δ*F*/*F* ± s.e.m.) at three different time points (left, week 3; middle, week 6 and right, week 10). **e**, Fraction of cells tuned at six different time points in two mice. The number of tuned neurons does not decline with time, suggesting intact response properties despite labeling by RV∆G. **f**, Tuning patterns at week 10 of 11 jGCaMP7s-expressing neurons showing clear preferred direction tuning, as well as the tuning pattern of an untuned neuron, representative of roughly half of imaged cells. **g**, Direction tuning patterns of six individual cells recorded at multiple time points (from week 1 to week 10). The top two cells became visible at week 1, the middle two appeared at week 2 and the bottom two cells appeared at week 3. Tuning patterns remain stable over the entire imaging period. **h**, Single-cell fluorescence time courses for 25 cells, showing activity over the first 120 s of visual stimulation. Cells are ranked in descending order of total activity. Scale bar: 10 s.

Finally, we conducted experiments designed to provide proof of concept for long-term functional imaging of synaptically connected networks of neurons (Fig. 6). We began by making a Flp-dependent jGCaMP7s reporter AAV and conducted pilot studies in PV-Cre mice to determine a dilution that would not itself cause mass cytotoxicity due to overexpression of jGCaMP7s (refs. ^28,44^) (Supplementary Fig. 3). We then conducted an experiment similar in design to the structural imaging study, except that the RV was mixed with the jGCaMP7s AAV before injection and headmount/coverslip implantation (Fig. 6a). We imaged labeled neurons at or near the injection sites over a series of sessions spanning 10 weeks. We imaged the jGCaMP7s signal while the awake mice viewed a series of drifting grating stimuli (Fig. 6b,c). We also imaged tdTomato fluorescence in the same fields of view to confirm that jGCaMP7s expression was restricted to RV-labeled neurons (Fig. 6c). As shown in Fig. 6d–h, as well as Supplementary Figs. 3–5, we observed clear orientation tuning curves in many of the labeled neurons, with visual responses appearing to remain stable across repeated imaging sessions over several months (Fig. 6d–h and Supplementary Fig. 5). Following the full course of two-photon imaging, we conducted postmortem sectioning and confocal imaging to confirm that viral spread had occurred in the imaged mice (Supplementary Fig. 6).

## Discussion

We have shown here that it is possible to use nontoxic double-deletion-mutant rabies viral vectors to label direct inputs to neurons targeted based on either their extrinsic projections or their gene expression (using Cre or Flp lines) and that use of the Tet-Off system results in relatively little toxicity even to the source cells.

A drawback of the second-generation system introduced here is its slow time course, necessitating multiweek incubation times (Figs. 1–3). We presume that this is due to low L expression from the TRE-CB allele given the low level of tTA expressed by the intentionally dilute Cre- or Flp-dependent helper virus^29,30^. This hypothesis is consistent with our inability to detect clear mCardinal signal by confocal microscopy (Extended Data Fig. 1) and may also explain the high percentage of surviving source cells that we found. While a slow time course is inconvenient, it may be the necessary price of preserving the source cells.

An interesting finding from the current study was that using an RV∆GL expressing Cre instead of one expressing Flpo resulted in higher mean numbers of labeled cells under some conditions (Figs. 1–3), although these comparisons were not statistically significant. Our working hypothesis is that such a difference, if real, could be due to a difference in the efficiency of the two recombinases^38,45,46^, with Cre more faithfully reporting the actual spread of the virus than Flpo when the recombinases are expressed at low levels (for example, by ∆GL RVs). A relative inefficiency of Flpo could also, however, cause lower efficiency of the Flp-dependent helper virus combination itself relative to the Cre-dependent one. This is consistent with the first-generation system’s lower efficiency in the Flp-dependent corticostriatal experiments than in the Cre-dependent ones (Fig. 1), as well as similarly in DAT-P2A-Flpo mice (Fig. 3) than in DAT-IRES-Cre mice (Fig. 2). In this study we simply matched the titers of the Flp-dependent combination to those used for the Cre-dependent one, but titration and further optimization of the Flp-dependent helper virus might improve performance.

We note that, even apart from the RV and its complementing gene products, several of the components of the system have the potential to cause cytotoxicity, including tTA^28,47,48^, GCaMPs^28,44^ and even AAV itself^49^. These can be expected to contribute to the toxicity to source cells, perhaps making it more remarkable that we found that so many of them survived, albeit with some perturbation to their membrane properties (Fig. 5).

Perhaps notably, our finding that ∆GL RVs do not spread when not complemented by L expression in *trans*, even when G is provided in *trans*, indicates that deletion of either L or G alone is sufficient to prevent transsynaptic spread. This suggests the possibility of a third-generation monosynaptic tracing system, based on single-deletion-mutant ∆L viruses^50^ complemented by expression in *trans* of just L.

## Methods

All experiments involving animals were conducted according to National Institutes of Health guidelines and approved by the MIT Committee for Animal Care. Mice were housed 1–5 per cage under a normal light/dark cycle, at 70 ± 2 °F and 30–70% humidity, for all experiments.

### Cloning

pAAV-syn-F14F15S-jGCaMP7s (Addgene, 178514) was made by cloning a Flp-dependent FLEX arrangement of mutually incompatible FRT sites^51^ into pAAV-synP-FLEX-EGFP-B19G (Addgene, 59333) followed by the jGCaMP7s (ref. ^52^) gene from pGP-CMV-jGCaMP7s (Addgene, 104463).

pAAV-syn-F14F15S-splitTVA-EGFP-tTA_v2 (Addgene, 183352) was made similarly but with the tricistronic open reading frame from pAAV-syn-FLEX-splitTVA-EGFP-tTA (Addgene, 100798) except for the use of FRT sites instead of lox ones (and with a 2-bp frameshift of the in-frame FRT site to prevent creation of a premature stop codon). Note that this plasmid is a replacement for pAAV-synP-F14F15S-splitTVA-EGFP-tTA, which was described in an earlier preprint of this manuscript.

pAAV-syn-FLEX-tTA (Addgene, 178516) was made by cloning a codon-optimized tet transactivator gene^53^ into pAAV-synP-FLEX-EGFP-B19G (Addgene, 59333).

pAAV-syn-Flpo (Addgene, 174378) and pAAV-syn-mCre (Addgene, 178515) were made by replacing the FLEX cassette from pAAV-synP-FLEX-EGFP-B19G (Addgene, 59333) with mouse-codon-optimized genes for Flp^38^ and Cre^36^.

pCAG-hypBase was made by synthesizing a fragment encoding the hyperactive piggyBac transposase iPB7 (ref. ^54^) and cloning it into the EcoRI and NotI sites of pCAG-GFP^55^ (Addgene, 11150).

The piggyBac plasmid pB-CAG-B19L-IRES-mCherry-WPRE-BGHpA (Addgene, 178518) was made by cloning the CAG promoter from pCAG-B19G (Addgene, 59921), the SAD B19 L gene, the EMCV IRES^56^, the mCherry^57^ gene and the woodchuck post-transcriptional regulatory element and bovine growth hormone polyadenylation signal from pCSC-SP-PW-GFP (Addgene, 12337) into PB-CMV-MCS-EF1-Puro (System Biosciences, PB510B-1).

The TLoop-style^58^ lentiviral transfer vectors pLV-TTBG (Addgene, 115232) and pLV-TTBL (Addgene, 115233) were made by replacing the CMV promoter and EGFP gene in pCSC-SP-PW-GFP (Addgene 12337) with the leaky tetracycline response element from pAAV-FLEX-hGTB (Addgene 26196) followed by a tricistronic open reading frame consisting of the genes for the improved tetracycline transactivator (itTA)^59^, mTagBFP2 (ref. ^35^) and either the glycoprotein or polymerase gene, respectively, of the RV SAD B19 strain, separated by P2A elements.

### Mouse strains

Mice used in this study were crosses (triple transgenic for DAT-IRES-Cre and PV-Cre experiments, double transgenic for corticostriatal experiments, double or single transgenic (either Cre-negative or L-negative) for control experiments) of the following strains, all in a C57BL/6J (Jackson Laboratory, 000664) background:

PV-Cre^43^, DAT-IRES-Cre^42^, Ai14 (ref. ^39^) and DAT-P2A-Flpo (ref. ^60^) were purchased from Jackson Laboratory (017320, 006660, 007914 and 035436).

The Flp-dependent tdTomato reporter line Ai65F was obtained in our case by crossing the Cre- and Flp-dependent tdTomato double-reporter line Ai65D (ref. ^27^) (Jackson Laboratory, 021875) to the Cre deleter line Meox2-Cre (ref. ^61^) (Jackson Laboratory, 003755) and then breeding out the Meox2-Cre allele. An equivalent Ai65F line, made using a different Cre deleter line, was described in ref. ^28^ and is now available from Jackson Laboratory (032864).

The L-expressing mouse line TRE-CB (TRE-tight-mCardinal-P2A-B19L, being distributed by Jackson Laboratory (036974)) was generated by cloning the genes for mCardinal^24^ and the RV (SAD B19) polymerase, separated by a picornavirus 2A element^62^, into Ai62(TITL-tdT) Flp-in replacement vector (Addgene, 61576), to make the Flp-in construct TRE-mCardinal-P2A-B19L Flp-in vector (Addgene, 178519). The lox-stop-lox sequence present in the original Flp-in vector was removed to make the new construct so that the tetracycline response element TRE-tight^63^ drives mCardinal-P2A-B19L directly with no dependence on Cre recombination. This plasmid was then used for FLP-mediated targeted insertion into the TIGRE locus^26^ in Ai99 embryonic stem cells as described^28^. Clones verified as containing the correct insert were used for the production of knock-in mice by the Koch Institute for Integrative Cancer Research at MIT. All subsequent breeding steps were with mice of a C57BL/6J background.

Strains, sexes and ages (at the time of injection) of mice used by the experiment were as follows (all in C57BL/6J background):

Corticostriatal tracing (Fig. 1):

Ai14 het—10F, 2M, age range 6–11 weeks

Ai65F het—7F, 1M, age range 14–28 weeks

Ai65F homo—2F, 2M, age 17 weeks

Ai14 x TRE-CB het/het—18F, 6M, age range 8–13 weeks

Ai65F x TRE-CB het/het—13F, 11M, age range 6–26 weeks

Tracing in Cre mice (Fig. 2):

Ai65F x TRE-CB het/het—5F, 11M, age range 7–17 weeks

DAT-Cre x Ai65F het/het—4F, 4M, age range 8–11 weeks

DAT-Cre x Ai65F x TRE-CB het/het/het—13F, 15M, age range 5–9 weeks

PV-Cre x Ai65F het/het—4F, 4M, age range 6–7 weeks

PV-Cre x Ai65F x TRE-CB het/het/het—19F, 9M, age range 6–22 weeks

Tracing in Flpo mice (Fig. 3):

Ai65F x TRE-CB het/het—3F, 1M, age 7 weeks

DAT-Flpo x Ai14 het/het—2F, 2M, age range 7–19 weeks

DAT-Flpo x Ai14 x TRE-CB het/het—3F, 9M, age range 6–13 weeks

Structural two-photon imaging (Fig. 4):

PV-Cre x Ai65F x TRE-CB het/het/het—6M, age range 15–20 weeks

Whole-cell electrophysiology (Fig. 5):

PV-Cre x Ai65F het/het—8F, 1M, age range 28–47 weeks

PV-Cre x Ai65F x TRE-CB het/het/het—3F, 1M, age 6 weeks

Functional two-photon imaging (Fig. 6):

PV-Cre x Ai65F x TRE-CB het/het/het—2M, age range 10–14 weeks

Two-photon imaging of RV∆G-Flpo and RV∆GL-Flpo (Supplementary Fig. 1):

Ai65F het—3F, 4M, age range 7–29 weeks

### Production of lentiviral vectors for making cell lines

Lentiviral vectors were made as described^64^ but with the VSVG expression vector pMD2.G (Addgene, 12259) as the envelope plasmid and with the following transfer vectors:

pLV-TTBL (described above), to make the L-expressing lentiviral vector LV-TTBL(VSVG),

pLV-U-TVA950 (Addgene, 115236), to make the TVA-expressing lentiviral vector LV-U-TVA950(VSVG).

### Production of cell lines

To make the L-expressing cell line BHK-B19L, BHK-21 cells (ATCC, CCL-10) were transfected with pCAG-hypBase and pB-CAG-B19L-IRES-mCherry-WPRE-BGHpA. The cells were expanded into two 15c plates, then resuspended and sorted on a BD FACS Aria to collect the brightest 5% of mCherry-expressing cells.

BHK-B19L-TVA950 cells were made by infecting BHK-B19L cells (described above) with LV-U-TVA950(VSVG) at a multiplicity of infection of approximately 100. Cells were then expanded and frozen in a medium containing 10% DMSO for subsequent use.

The BHK-EnvA2-TTBL2 cell line, expressing both the EnvARGCD fusion protein^1^ and the RV polymerase, was made by infecting BHK-EnvA2 cells^65^ with the L-expressing lentiviral vector LV-TTBL(VSVG, described above) at a multiplicity of infection of approximately 100. The cells were expanded into three 15 cm plates and then sorted on a BD FACS Aria running BD FACSDiva v.9.0 to collect those cells with the highest 2% of both green (EnvA) and blue (L) fluorescence. After expanding the collected cells again, we observed that blue fluorescence was so dim as to be difficult to detect by eye. We, therefore, sorted the cells a second time, again retaining cells with the brightest 2% fluorescence in both channels. The twice-sorted cells, now called BHK-EnvA2-TTBL2, were expanded again and frozen in a medium containing 10% DMSO for subsequent use.

### Production and titering of adeno-associated viruses

AAV genome plasmids pAAV-syn-FLEX-splitTVA-EGFP-tTA (Addgene, 100798) and pAAV-TREtight-mTagBFP2-B19G (Addgene, 100799) have been described previously^19^. These genomes were packaged in serotype 1 AAV capsids by Addgene (52473-AAV1, 100798-AAV1 and 100799-AAV1). The two helper AAVs from Addgene were diluted in Dulbecco’s phosphate-buffered saline (DPBS; Thermo Fisher Scientific, 14190-250) to final titers of 7.22 × 10^10^ gc ml^−1^ for AAV1-syn-FLEX-splitTVA-EGFP-tTA and 6.50 × 10^11^ gc ml^−1^ for AAV-TREtight-mTagBFP2-B19G, then combined in a 50/50 ratio by volume before injection. AAVs from Addgene were initially thawed and aliquoted in 20× (5 µl) aliquots. To make working dilutions, a 5 µl aliquot of undiluted virus was thawed and diluted in DPBS (Thermo Fisher Scientific, 14190-250) to the desired working dilutions (1:20 or 1:200)^29,30^. pAAV-syn-FLEX-tTA (described above) was packaged as serotype 1 by the UNC Vector Core with titer of 2.51× 10^12^ gc ml^−1^.

rAAV2-retro-syn-Cre, rAAV2-retro-syn-Flpo, AAV1-syn-F14F15S-jGCaMP7s and AAV1-syn-F14F15S-sTpEptTA_v2 were made by transfecting 8x15c plates (per virus) of HEK 293T cells using Xfect transfection reagent (Takara, 631318). Briefly, plates treated with poly-l-lysine solution were transfected with pAAV vector, pHelper (Cell Biolabs, VPK-421), and the appropriate rep/cap plasmid (rAAV2-retro helper (Addgene, 81070) or pAAV-RC1 (Cell Biolabs, VPK-421)). Four hours after transfection, the medium from plates was aspirated and replaced with 15 ml DMEM (Thermo Fisher Scientific, 11995-081) containing 2% fetal bovine serum (FBS; HyClone, SH30071.02) and antibiotic/antimycotic (Thermo Fisher Scientific, 15240-062). Seventy-two hours after transfection, the supernatants were collected from each plate and replaced again with 15 ml DMEM with 2% FBS and antibiotic/antimycotic. Supernatants were stored in 50 m; tubes at 4 °C. One hundred and twenty hours after transfection, cells and supernatants were collected and transferred into sterile 50 ml tubes. Cells were pelleted by centrifugation and washed with DPBS, and supernatants were transferred into a sterile bottle for polyethylene glycol (PEG) precipitation. Cells were lysed by four freeze/thaw cycles and stored at −80 °C until the final thaw and purification steps. In total, 50 ml of cold 40% PEG 8000 in 2.5 M NaCl was added to 250 ml of clarified supernatants and mixed thoroughly. This solution was incubated at 4 °C overnight, then transferred into sterile 50 ml tubes and centrifugated at 4,000*g* for 30 min at 4 °C. Supernatants were discarded and pellets were resuspended in a total of 1 ml (150 mM) NaCl and 50 mM Tris buffer. For the final purification steps, cell pellets were combined with PEG-precipitated virus, treated with Benzonase nuclease (250 U µl^−1^) for 30 min at 37 °C and then centrifugated at 7,188*g* for 30 min at 4 °C. The clarified lysate was transferred to Optiseal tubes (Beckman, 361625) containing layered gradients of iodixanol (15%, 25%, 40% and 54% iodixanol solutions). Tubes were ultracentrifugated in a Beckman 70 Ti fixed-angle rotor at 70,000 r.p.m. (217,000*g* at minimum radius and 505,000*g* at maximum radius) for 1 h and 40 min at 15 °C on a WX 80+ centrifuge (Thermo Fisher Scientific). The layers containing the virus were carefully removed from between the 40% and 54% iodixanol layers, filtered through 0.22 µm polyethersulfone membrane syringe filters and concentrated in Amicon Ultra-15 Centrifuge Units (Millipore, UFC910008), with three full buffer exchanges with DPBS to remove all residual iodixanol. Each batch of the virus was concentrated to a final volume of 250–300 µl total, aliquoted in 5 µl aliquots, stored at −80 °C and titered by quantitative PCR.

### Production of RVs

EnvA-enveloped ∆G RVs were made as described previously^11,65,66^, using genome plasmids pRV∆G-4Flpo (Addgene, 122050) and pRV∆G-4Cre (Addgene, 98034).

EnvA-enveloped ∆GL RVs were rescued in 15 cm plates as described^4^ using genome plasmids pRV∆GL-4Flpo (Addgene, 98040) and pRV∆GL-4Cre (Addgene, 98039). Supernatants from rescue plates were passaged on 15 cm plates of HEK 293T/17 cells (ATCC) transfected with pLV-TTBG and pLV-TTBL (described above) using Xfect transfection reagent (Takara, 631318) according to the manufacturer’s protocol. Supernatants were titered on reporter cells^4^ and used to infect BHK-EnvA2-TTBL2 cells (described above) at a multiplicity of infection of approximately two. Supernatants from these producer cells were collected and purified as described^65,66^.

### Titering of RVs

Recombinase-expressing RVs were titered primarily on the reporter cell lines 293T-FLEX-BC, 293T-F14F15S-BC, 293T-FLEX-BC-TVA and 293T-F14F15S-BC-TVA as described^11^ but using threefold serial dilutions, instead of tenfold dilutions, for higher precision. For the corticostriatal experiment, to directly compare viral titers in a way that did not depend on the efficacy of the encoded recombinases, we made the BHK-B19L-TVA950 cell line (described above), which expresses both TVA, to allow infection by EnvA-enveloped viruses, and L, to allow intracellular replication of ∆GL viruses so that infected cells could be clearly labeled using immunostaining against the RV nucleoprotein. BHK-B19L-TVA950 cells, as well as BHK-B19L cells, were infected with threefold serial dilutions of RV∆GL-Cre(EnvA) and RV∆GL-Flpo(EnvA. Three days after infection, cells were fixed with 4% paraformaldehyde in PBS and immunostained with a FITC-conjugated anti-nucleocapsid monoclonal antibody blend (Light Diagnostics Rabies DFA, EMD Millipore, 5100) diluted 1:100 in 1% BSA and 0.1% Triton in PBS. Immunolabeled cells were analyzed by flow cytometry to determine titers as described^65^.

The titers of the RVs were determined to be as follows:

RV∆GL-Cre(EnvA):

on 293T-FLEX-BC-TVA950: 1.28 × 10^9^ iu ml^−1^

on 293T-FLEX-BC (no TVA): 1.48 × 10^5^ iu ml^−1^

on BHK-B19L-TVA950 with anti-N staining: 6.13 × 10^8^ iu ml^−1^

RV∆GL-Flpo(EnvA):

on 293T-F14F15S-BC-TVA950: 2.94 × 10^8^ iu ml^−1^

on 293T-F14F15S-BC (no TVA): 3.26 × 10^5^ iu ml^−1^

on BHK-B19L-TVA950 with anti-N staining:7.67 × 10^8^ iu ml^−1^

RV∆G-Cre(EnvA):

on 293T-FLEX-BC-TVA950: 1.24 × 10^10^ iu ml^−1^

on 293T-FLEX-BC (no TVA): 4.02 × 10^5^ iu ml^−1^

RV∆G-Flpo(EnvA):

on 293T-F14F15S-BC-TVA950: 4.49 × 10^9^ iu ml^−1^

on 293T-F14F15S-BC (no TVA): 4.81 × 10^5^ iu ml^−1^

RV∆G-4Flpo(B19G):

on 293T-F14F15S-BC: 1.86 × 10^10^ iu ml^−1^

RV∆GL-Flpo(B19G):

on 293T-F14F15S-BC: 1.86 × 10^9^ iu ml^−1^

### Stereotaxic injections

Stereotaxic injections were made in anesthetized adult mice of both sexes using a stereotaxic instrument (Stoelting, 51925) and a custom injection apparatus consisting of a hydraulic manipulator (Narishige, MO-10) with headstage coupled via custom adaptors to a wire plunger advanced through pulled glass capillaries (Drummond, Wiretrol II) back-filled with mineral oil and front-filled with viral vector solution. We have described this injection system in detail previously^29^.

For the corticostriatal experiments (Fig. 1), 200 nl of AAV2-retro-syn-Flpo (1.16 × 10^13^ GC ml^−1^; Ai14 or Ai14 x TRE-CB mice) or AAV2-retro-synP-mCre (1.48 × 10^13^ GC ml^−1^, diluted to 1.16 × 10^13^ GC ml^−1^ for matching to the Flpo version; Ai65F or Ai65F x TRE-CB mice) was injected into dorsolateral striatum (anteroposterior (AP) = +0.74 mm with respect to (w.r.t.) bregma, lateromedial (LM) = 2.25 mm w.r.t. bregma, dorsoventral (DV) = −2.30 mm w.r.t the brain surface). In the same surgery, 250 nl of helper virus mixture (AAV1-syn-F14F15S-sTpEptTA_v2 (diluted to 7.19 × 10^10^ GC ml^−1^; Ai14 or Ai14 x TRE-CB mice) or AAV1-syn-FLEX-splitTVA-EGFP-tTA (diluted to 7.22 × 10^10^ GC ml^−1^; Ai65F or Ai65F x TRE-CB mice) mixed with AAV1-TREtight-mTagBFP2-B19G (diluted to 6.50 × 10^11^) in a 50/50 ratio by volume) was injected into S1BF, layer 5 DLS projection region (AP = −1.55 mm w.r.t. bregma, LM = 3.00 mm w.r.t. bregma, DV = −0.75 mm w.r.t the brain surface). Seven days after AAV injection, 250 nl of RV∆GL-Cre(EnvA; 6.13 × 10^8 ^iu ml^−1^ as titered by nucleoprotein staining on L-expressing cells; see above) or RV∆GL-Flpo(EnvA; 7.67 × 10^8^ iu ml^−1^, diluted to 6.13 × 10^8^ iu ml^−1^) or RV∆G-Flpo(EnvA; 4.49 × 10^9^ iu ml^−1^) or RV∆G-Cre(EnvA; 1.24 × 10^10^ iu ml^−1^, diluted to 4.49 × 10^9^ iu ml^−1^) was injected at the same cortical injection site as the helper viruses. For no-G controls, DPBS was included instead of AAV1-TREtight-mTagBFP2-B19G.

For the anatomical experiments in Cre lines (Fig. 2), 300 nl of helper AAV mixture (AAV1-syn-FLEX-splitTVA-EGFP-tTA (diluted to 7.22 × 10^10^ GC ml^−1^) mixed with AAV1-TREtight-mTagBFP2-B19G (diluted to 6.50 × 10^11^ GC ml^−1^) in a 50/50 ratio by volume; note that these are the same dilutions as we have recommended previously^29,30^ but retitered by qPCR in our own lab) was injected into either primary somatosensory cortex or SNc (see below for stereotaxic coordinates). Injection coordinates for SNc were as follows: AP = −3.00 mm w.r.t. bregma, LM = +1.50 mm w.r.t bregma, DV = −4.20 mm w.r.t the brain surface). Injection coordinates for somatosensory cortex were as follows: AP = −0.58 mm w.r.t. bregma, LM = 3.00 mm w.r.t. bregma, DV = −1.00 mm w.r.t the brain surface. Seven days after AAV injection, 500 nl (PV-Cre and DAT-IRES-Cre) of RV∆GL-Flpo(EnvA; 7.67 × 10^8^ iu ml^−1^) was injected at the same injection site as the AAVs. The experiments in DAT-P2A-Flpo mice (Fig. 3) were done similarly but using the Flp-dependent AAV-syn-F14F15S-EGFP-tTA_v2 (diluted to 7.19 × 10^10^ GC ml^−1^) and RV∆G-Cre(EnvA; 1.24 × 10^10^ iu ml^−1^, diluted to 4.49 × 10^9^ iu ml^−1^).

For longitudinal two-photon imaging for comparison of RV∆G-Flpo(B19G) and RV∆GL-Flpo(B19G), 200 nl RV∆G-Flpo(B19G; 1.86 × 10^10^ iu ml^−1^) or RV∆GL-Flpo(B19G; 1.86 × 10^9^ iu ml^−1^) was injected into V1 (AP = −2.70 mm w.r.t. bregma, LM = 2.50 mm w.r.t. bregma, DV = −0.26 mm w.r.t. brain surface) into Ai65F mice. Glass windows composed of a 3 mm-diameter glass coverslip (Warner Instruments CS-3R) glued (Optical Adhesive 61, Norland Products) to a 5 mm-diameter glass coverslip (Warner Instruments CS-5R) were then affixed over the craniotomy with Metabond (Parkell), and custom stainless steel headplates (eMachineShop) were affixed to the skulls around the windows.

For longitudinal two-photon structural imaging of live monosynaptic tracing at the injection site, a 3 mm craniotomy was opened over primary visual cortex (V1). In total, 300 nl of AAV1-syn-FLEX-splitTVA-EGFP-tTA (diluted to 7.22 × 10^10^ GC ml^−1^; Ai65F or Ai65F x TRE-CB mice) mixed with AAV1-TREtight-mTagBFP2-B19G (diluted to 6.50 × 10^11^) in a 50/50 ratio by volume was injected into V1 (AP = −2.70 mm w.r.t bregma, LM = 2.50 mm w.r.t. bregma, DV = −0.26 mm w.r.t. brain surface), followed by implantation of windows as described above. Seven days after the injection of helper AAVs, the coverslips were removed and 100 nl of RV∆GL-Flpo (EnvA; 7.67 × 10^8^ iu ml^−1^) was injected at the same site. Coverslips were reapplied and custom stainless steel headplates (eMachineShop) were affixed to the skulls around the windows.

For functional imaging experiments, the V1 injection coordinates were AP = −2.45 mm w.r.t bregma, LM = 2.00 mm w.r.t bregma, DV = −0.26 mm w.r.t. brain surface, and the RV∆GL-Flpo(EnvA; 7.67 × 10^8^ iu ml^−1^) was mixed in a 50/50 ratio by volume with AAV1-syn-F14F15S-jGCaMP7s (5.44 × 10^12^ GC ml^−1^, diluted 1:10 in DPBS) before injection of 200 nl of the mixture 7 d following the helper AAV injection.

### Doxycycline administration

‘No-dox’ mice were fed with regular rodent chow throughout, while ‘dox (food)’ mice were switched to chow containing doxycycline 200 mg kg^−1^ (Thermo Fisher Scientific, 14-727-450) beginning 2 weeks after RV injection and maintained on doxycycline chow until perfusion, to suppress rabies viral polymerase and glycoprotein expression. ‘Dox (injection + food)’ mice, including those used for the structural two-photon imaging experiment, also received intraperitoneal injections of 100 mg kg^−1^ doxycycline every 12 h for 3 d, beginning 2 weeks after RV injection.

### In vivo two-photon imaging and image analysis

For longitudinal two-photon imaging of live monosynaptic tracing, injection sites were imaged on a Prairie/Bruker Ultima IV In Vivo two-photon microscope driven by a Spectra-Physics Mai-Tai Deep See laser with a mode-locked Ti:sapphire laser emitting at a wavelength of 1,020 nm for tdTomato. Mice were reanesthetized and mounted via their headplates to a custom frame, with ointment applied to protect their eyes and with a hand warmer maintaining body temperature. One field of view was chosen in each mouse in the area of maximal fluorescent labeling. The imaging parameters were as follows: image size 1,024 × 1,024 pixels (565.1 μm × 565.1 μm), 0.360 Hz frame rate, dwell time 2.0 μs, ×1 optical zoom and z-stack step size 1 μm. Image acquisition was controlled with Prairie View 5.4 software. Laser power exiting the 20× water-immersion objective (Zeiss, W plan-apochromat, NA 1.0) varied between 20 and 65 mW depending on focal plane depth (Pockels cell value was automatically increased from 450 at the top section of each stack to 750 at the bottom section). For the example images of labeled cells, maximum intensity projections (stacks of 150–400 μm) were made with Fiji software. Cell counting was performed with the ImageJ Cell Counter plugin. When doing cell counting, week 1 tdTomato-labeled cells were defined as a reference; the remaining week 1 cells were the same cells at a later time point that aligned with week 1 reference cells but the not-visible cells at week 1 (the dead cells). Plots of cell counts were made with Origin 7.0 software (OriginLab) or Prism 9 (GraphPad Software).

For functional two-photon imaging of source cells, injection sites in left-hemisphere V1 were chosen as the imaging area. This imaging was performed using the same microscope (5.356-Hz frame rate, 1,024 × 128 pixels (565.1 μm × 565.1 μm), dwell time 0.8 μs, ×1 optical zoom and scan angle 45°) with the same objective and laser (at 920 nm) as in the structural imaging experiments. Laser power at the objective ranged from 10 to 65 mW. Calcium imaging data were acquired in supragranular layers (100–200 μm deep). The animal was awake, head-fixed and the animal body was free to lay into a customized tube with some holes for air circulation. No behavioral training or reward was given. To find the same FOVs in subsequent weeks, surface vasculature map was drawn on a notebook for each mouse under a microscope with light source (Leica M50), and an asterisk marker was put near the location of FOVs in this map for reference when doing the first imaging session. The cell body images from averaged data taken in the first session provided a template for fine alignment. Visual stimuli were generated in MATLAB (R2015R version) with custom software based on Psychtoolbox 3.0.17 (http://psychtoolbox.org) and shown on the same liquid crystal display screen as in the widefield mapping experiments. Each condition consisted of 2 s of a full-field sine-wave grating drifting in one direction, presented at 80% contrast with spatial frequency of 0.04 cycles/degree, followed by 2 s of uniform mean luminance (gray). All permutations of 12 directions (30° steps) and five temporal frequencies (TFs, 1, 2, 4, 8 and 15 Hz) were shown, in randomized order. The complete set was repeated ten times, for a total stimulation period of 40 min per FOV per session. Cells were then manually segmented, and single-cell fluorescence traces were extracted by averaging the fluorescence of all pixels masking the soma by ImageJ (versions: 2-0-0-rc-69) software. The mean Δ*F*/*F* over the full 2 s of each stimulus condition was used to calculate orientation tuning curves, with background fluorescence (*F*) in Δ*F*/*F* taken as the value of the trace immediately preceding a condition, averaged over all conditions. The raw calcium traces from cells within individual FOVs (not across FOVs, given different imaging conditions across animals and time points) were sorted by mean fluorescence. For ‘tuned’ cells in Fig. 4e, the counts are based on all imaged neurons’ individual tuning curves, plotted in MATLAB. Any cell showing response to a preferred orientation (including narrowly tuned neurons and broadly tuned neurons) at any temporal frequency (1 Hz, 2 Hz, 4 Hz, 8 Hz or 15 Hz) was counted manually as a tuned cell (see Supplementary Table 7 for counts of tuned and untuned cells).

### Brain slice electrophysiology

Coronal brain slices containing the S1 were collected from 12- to 32-week-old PV-Cre x Ai65F x TRE-CB het/het/het (∆GL group) or PV-Cre x Ai65F het/het (∆G and AAV-only groups) for ex vivo patch-clamp recordings. Mice were anesthetized with isoflurane, perfused with an ice-cold cutting solution and decapitated using scissors. Brains were extracted and immersed in ice-cold (0–4 °C) sucrose cutting solution containing 252 mM sucrose, 26 mM NaHCO_3_, 2.5 mM KCl, 1.25 mM NaH_2_PO_4_, 1 mM CaCl_2_, 5 mM MgCl_2_ and 10 mM glucose, which was oxygenated with 95% O_2_ and 5% CO_2_. The brains were trimmed, and coronal brain slices (300 µm) were sectioned using a vibratome (VT1200, Leica). After sectioning, slices for patch-clamp recordings were transferred to a holding chamber containing oxygenated patch-clamp recording medium (aCSF) containing 126 mM NaCl, 2.5 mM KCl, 1.25 mM NaH_2_PO_4_, 1.3 mM MgCl_2_, 2.5 mM CaCl_2_, 26 mM NaHCO_3_ and 10 mM glucose, where they were maintained at 32 °C for 30 min before decreasing the chamber temperature to ~20 °C. Slices were transferred one at a time from the holding chamber to a submerged recording chamber mounted on the fixed stage of an Olympus BX51WI fluorescence microscope equipped with differential interference contrast illumination. The slices in the recording chamber were continuously perfused at a rate of 2 ml min^−1^ with recording aCSF at room temperature and continuously aerated with 95% O_2_/5% CO_2_. Whole-cell patch-clamp recordings were performed in the fluorescently labeled PV+ neurons and principal neurons in the S1 and neighboring S2 regions. The presynaptic principal neurons to PV cells in the AAV control group were randomly selected based on the size, morphology and accommodating firing pattern. Glass pipettes with a resistance of 4-8 MΩ were pulled from borosilicate glass (ID 1.2 mm, OD 1.65 mm) on a horizontal puller (Sutter P-97) and filled with an intracellular patch solution containing 130 mM potassium gluconate, 10 mM HEPES, 10 mM phosphocreatine Na_2_, 4 mM Mg-ATP, 0.4 mM Na-GTP, 5 mM KCl and 0.6 mM EGTA; pH was adjusted to 7.25 with KOH and the solution had a final osmolarity of 293 mOsm. Series resistance was continuously monitored. Cells were discarded when the series resistance changed more than 20%. Data were acquired using a Multiclamp 700B amplifier, a Digidata 1440A analog/digital interface and pClamp 10 software (Molecular Devices). Data were analyzed with Clampfit 10 (Molecular Devices). The membrane capacitance (Cm) and input resistance (Rm) were calculated from a membrane seal test conducted in voltage-clamp mode, in which 100-ms, 5-mV voltage steps were delivered at a frequency of 5 Hz. To evaluate the action potential rheobase, a 1-s, positive current was delivered in current-clamp mode at 10 pA steps. Rheobase was defined as the minimum current required to depolarize the membrane potential for action potential firing. Finally, to evaluate the relationship between injected current and firing frequency, we delivered a series of 0.5 s current pulses in the current clamp at 50 pA steps (50–250 pA for principal cells and 50–450 pA for PV cells). Statistical comparisons were conducted with unpaired Student’s *t* test or with a one- or two-way analysis of variance (ANOVA) followed by a post hoc Dunnett’s test as appropriate (*P* < 0.05 with a two-tailed analysis was considered significant).

### Perfusions and histology

One to twelve weeks (depending on the experiment; see main text) after injection of RV, mice were transcardially perfused with 4% paraformaldehyde in phosphate-buffered saline. Brains not used for two-photon imaging were postfixed overnight in 4% paraformaldehyde in PBS on a shaker at 4 °C and cut into 50 µm coronal (S1 injections) or parasagittal (SNc injections) sections on a vibrating microtome (Leica, VT-1000S). Sections were collected sequentially into six tubes containing cryoprotectant so that each tube contained a sixth of the collected tissue. For coronal sections, 15 rounds of sections were collected. For sagittal sections, 12 rounds were collected. Brains from mice used for two-photon imaging were postfixed in 4% paraformaldehyde/30% sucrose in PBS for 2 d on a shaker at 4 °C and then cut into 50 µm sections on a freezing microtome in a plane approximately tangential to the surface of the brain at the imaged location. Sections were immunostained as described^29^ with the following primary antibodies (as applicable) at the following respective dilutions: chicken anti-GFP (Aves Labs, GFP-1020; 1:500), guinea pig anti-parvalbumin (Synaptic Systems, 195004; 1:1,000), sheep anti-tyrosine hydroxylase (Millipore, AB1542; 1:1,000), with secondary antibodies donkey anti-chicken Alexa Fluor 488 (Jackson ImmunoResearch, 703-545-155; 1:200), donkey anti-guinea pig, Alexa Fluor 647 conjugated (Jackson Immuno Research 706-605-148; 1:200) and donkey anti-sheep, Alexa Fluor 647 conjugated (Jackson ImmunoResearch 713-605-147; 1:200). Sections were mounted with Prolong Diamond Antifade mounting medium (Thermo Fisher Scientific, P36970) and imaged on a confocal microscope (Zeiss, LSM 900).

### Cell counts and statistical analysis

Labeled neurons in striatum (in DAT-IRES-Cre mice), contralateral cortex and thalamus (in PV-Cre mice and for the corticostriatal experiments) and at the injection sites were counted either manually or with the automated Cell Counter function in ImageJ, in every sixth 50-µm section on an epifluorescence microscope (Zeiss Imager.Z2). Coronal sections (corticostriatal experiments and PV-Cre) included sections between 1.2 mm and −3.3 mm relative to bregma. Sagittal sections (DAT-IRES-Cre) covered sections 3.6 mm to 0.0 mm relative to Bregma. Cells expressing EGFP, mTagBFP2 or both, along with tdTomato, were counted by adding separate labels to each and then looking for overlapping cells. Groups were compared using single-factor ANOVAs in Microsoft Excel for Mac version 16.42. Symbols denoting significance levels on graphs are as follows: ***P < 0.001; **0.001 ≤ *P* < 0.01; *0.01 ≤ *P* < 0.05; not significant = *P* ≥ 0.05. Data distribution was assumed to be normal, but this was not formally tested. No statistical methods were used to predetermine sample sizes, but our sample sizes are similar to those reported in previous publications^50^ and are as large as practical given the large number of conditions tested.

#### Randomization

The data collection was not randomized. Animals were assigned to the various experimental groups based on availability. There was no randomization in the organization of the experimental conditions. Data collection and analysis were not performed blind to the conditions of the experiments. No animals or data points were excluded from the analysis.

### Reporting summary

Further information on research design is available in the Nature Portfolio Reporting Summary linked to this article.

## Online content

Any methods, additional references, Nature Portfolio reporting summaries, source data, extended data, supplementary information, acknowledgements, peer review information; details of author contributions and competing interests; and statements of data and code availability are available at 10.1038/s41593-023-01545-8.

### Supplementary information

Supplementary Information Supplementary Figs. 1–7.

Reporting Summary

Supplementary Table 1 Counts and statistics for corticostriatal experiments.

Supplementary Table 2 Counts and statistics for DAT-IRES-Cre experiments.

Supplementary Table 3 Counts and statistics for PV-Cre experiments.

Supplementary Table 4 Counts and statistics for DAT-P2A-Flpo experiments.

Supplementary Table 5 Counts for ∆G-Flpo and ∆GL-Flpo comparison.

Supplementary Table 6 Counts and calculations for estimation of starting cell survival rates.

Supplementary Table 7 Counts of tuned and untuned cells in functional imaging experiments.

## Data Availability

All cell counts and statistical analyses are provided in Supplementary Information. The new plasmids described in this paper have been deposited with Addgene with the accession given in Methods. The TRE-CB mouse line is available from the Jackson Laboratory (accession 036974).
