# Publisher Correction: Long-term labeling and imaging of synaptically connected neuronal networks in vivo using double-deletion-mutant rabies viruses

**DOI:** 10.1038/s41593-024-01584-9

**Published:** 2024-01-24

**Authors:** Lei Jin, Heather A. Sullivan, Mulangma Zhu, Thomas K. Lavin, Makoto Matsuyama, Xin Fu, Nicholas E. Lea, Ran Xu, YuanYuan Hou, Luca Rutigliani, Maxwell Pruner, Kelsey R. Babcock, Jacque Pak Kan Ip, Ming Hu, Tanya L. Daigle, Hongkui Zeng, Mriganka Sur, Guoping Feng, Ian R. Wickersham

**Affiliations:** 1 McGovern Institute for Brain Research, Massachusetts Institute of Technology, Cambridge, MA USA; 2 Department of Brain and Cognitive Sciences, Massachusetts Institute of Technology, Cambridge, MA USA; 3 Picower Institute for Learning and Memory, Massachusetts Institute of Technology, Cambridge, MA USA; 4 Allen Institute for Brain Science, Seattle, WA USA; 5 Stanley Center for Psychiatric Research, Broad Institute of MIT and Harvard, Cambridge, MA USA; 6 Present Address: Lingang Laboratory, Shanghai, China; 7 Present Address: School of Biomedical Sciences, The Chinese University of Hong Kong, Hong Kong, China; 8 Present Address: Department of Neuroscience, Baylor College of Medicine, Houston, TX USA

**Keywords:** Molecular neuroscience, Neural circuits

Correction to: *Nature Neuroscience* 10.1038/s41593-023-01545-8, published online 11 January 2024.

In the version of the article initially published, in Figs. 1–6 the minus sign was missing from “Day −7” and has now been inserted in the HTML and PDF versions of the article.

