# Supplementary material for: Long-term labeling and imaging of synaptically connected neuronal networks in vivo using double-deletion-mutant rabies viruses
Source: Nat Neurosci. 2024 Jan 11;27(2):373–83. doi: 10.1038/s41593-023-01545-8 (PMC10849964; doi:10.1038/s41593-023-01545-8)
Supplement: Supplementary file 1 — Supplementary Figs. 1–7. [file 41593_2023_1545_MOESM1_ESM.pdf]

nature neuroscience

Technical Report https://doi.org/10.1038/s41593-023-01545-8

Long-term labeling and imaging of synaptically connected neuronal networks in vivo using double-deletion-mutant rabies viruses

In the format provided by the authors and unedited

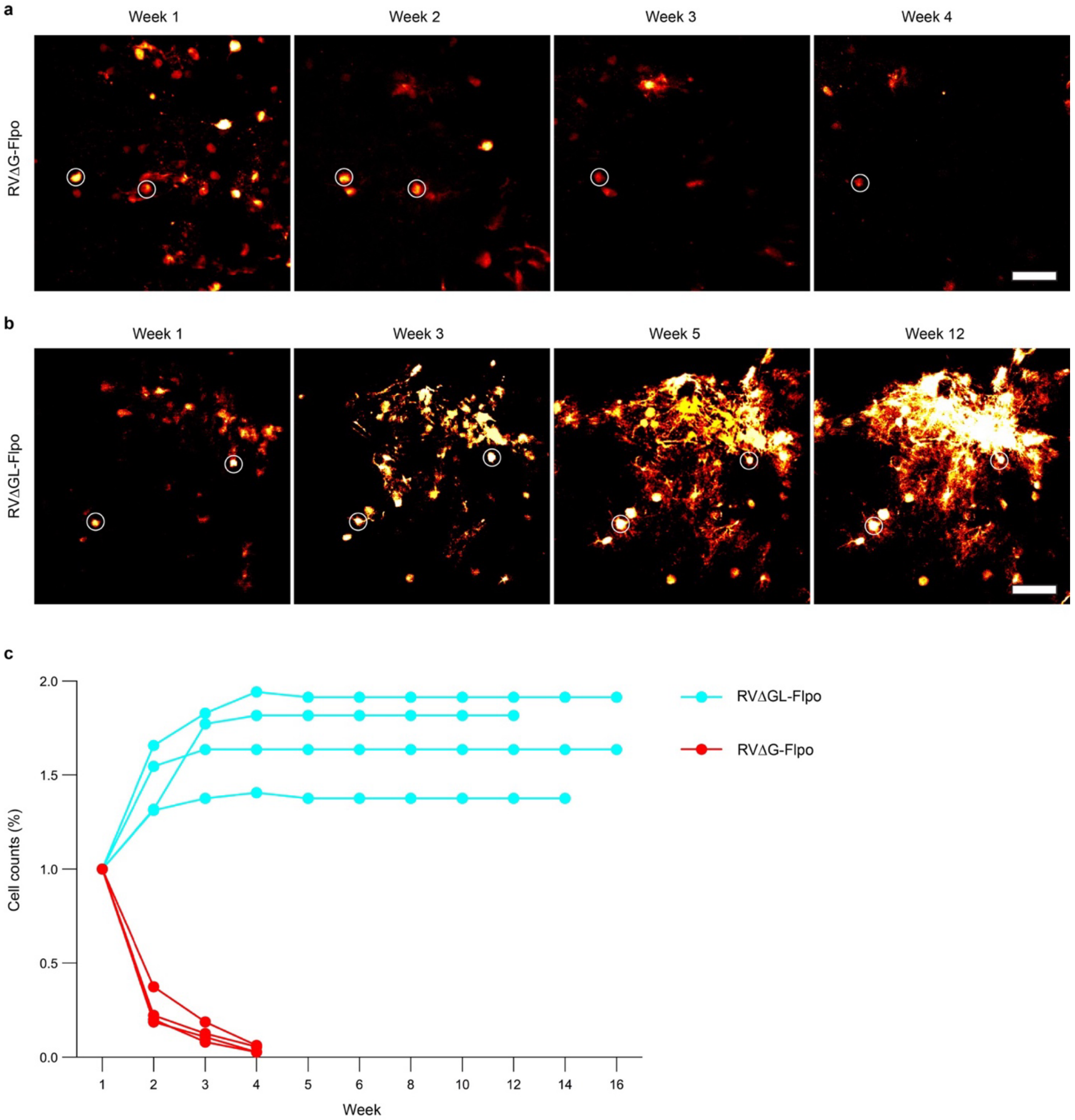

Supplementary Fig. 1: Second-generation rabies virus encoding Flpo does not kill most labeled neurons for at least 16 weeks. a, Representative images of longitudinal two-photon structural imaging fields of view (FOVs) from one week (left) to 4 weeks (right) after injection of a first-generation rabies viral vector encoding Flpo (RVΔG-Flpo). The viruses in this experiment were coated with the native RV glycoprotein for direct (TVA-independent) infection of neurons. Images are of the same FOV at different time points. This first-generation virus killed almost all infected cells in this FOV within 4 weeks: only two labeled cells (circled) survived until the 4-week timepoint. Scale bar: 100 μm. **b,** Representative images of longitudinal two-photon structural imaging FOVs from one week (left) to 12 weeks (right) after injection of a second-generation rabies viral vector encoding Flpo (RVΔGL-Flpo). Images are of the same FOV at different time points. All labeled cells are still present at 16 weeks, the last timepoint of imaging. Two example labeled cells are circled as fiducial markers. Scale bar: 100 μm. Images from **a-b** are representative of four independent experiments that yielded similar results. **c,** Fraction of visible labeled cells over time, relative to the number visible at one week after RVΔG-Flpo or RVΔGL-Flpo injection; connected sets of dots represent counts obtained from the same FOV within the same mouse at the different time points (for RVΔG-Flpo: 1 week to 4 weeks; for RVΔGL-Flpo: 1 week to 16 weeks). Cells infected by RVΔG-Flpo (red) have almost entirely disappeared by 4 weeks postinjection. The number of cells labeled by RVΔGL-Flpo (cyan) increases up to 4 weeks postinfection and then remains constant for as long as the brains were imaged.

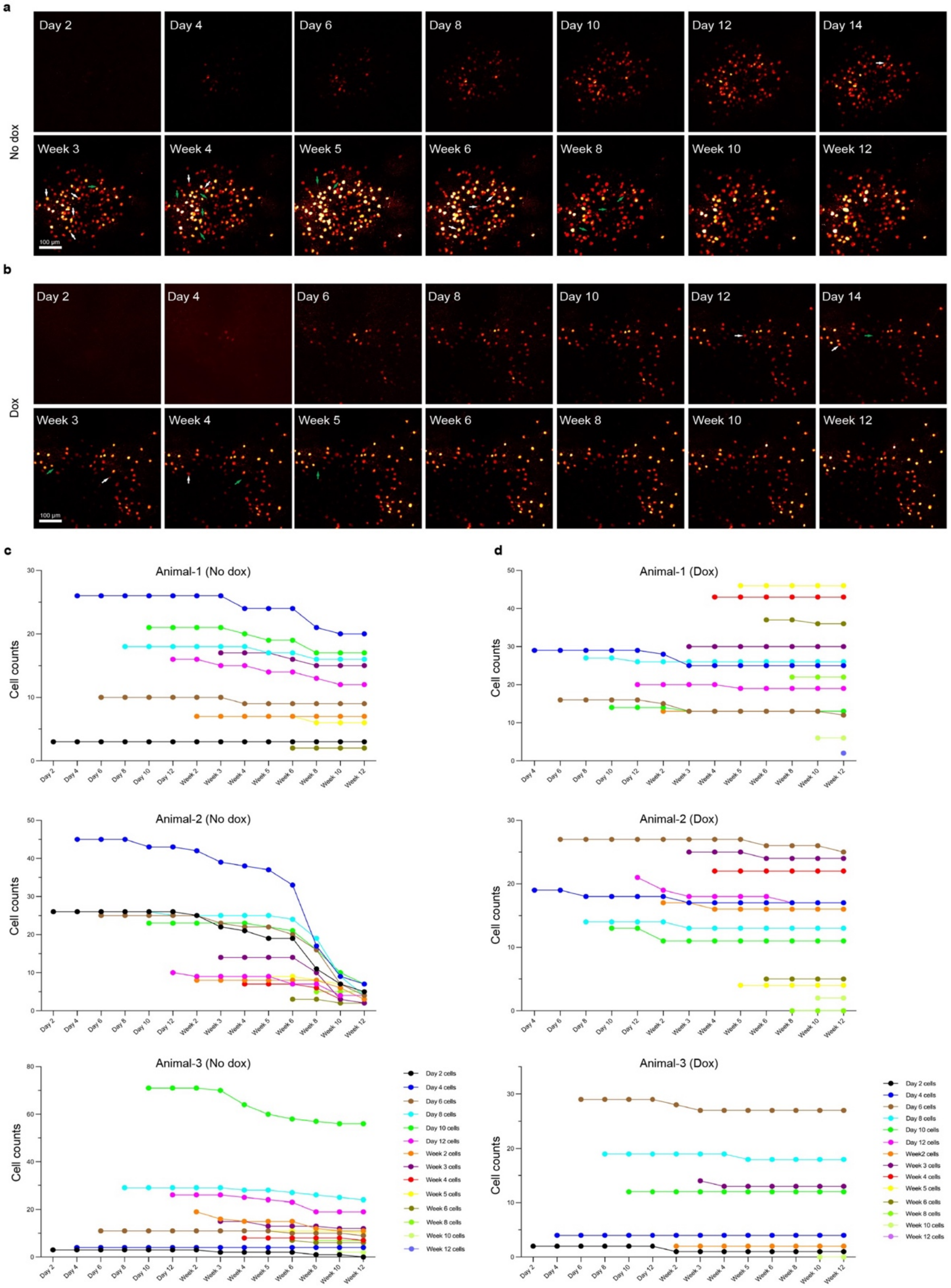

Supplementary Fig. 2: Additional examples of longitudinal two-photon imaging of second-generation monosynaptic tracing *in vivo*. a-b, Example images of tdTomato-labelled neurons at the

injection site in V1 over all imaging sessions, from 2 days to 12 weeks, in two mice. Images from **a-b** are representative of three independent experiments that yielded similar results. The ‘No dox’ mice (**a**) were fed with regular food throughout, while the ‘Dox’ mice (**b**) were fed with food containing doxycycline (200 mg/kg) starting at two weeks after RV injection until perfusion at week 12, in order to suppress expression of the rabies virus polymerase and glycoprotein genes. White arrows show example cells that are last seen at that time point, with green arrows indicating the former positions of those now-missing cells at the next time point. In these examples, a total of 20 out of 141 cells were lost in the ‘No dox’ mouse, whereas 5 out of 98 cells were lost in the ‘Dox’ mouse. Scale bar: 100 µm. **c-d,** Counts of tdTomato-labeled cells appearing at each timepoint in individual mice. Each connected set of dots represents the numbers of the cells that appeared at one timepoint that are still present at the subsequent timepoints.

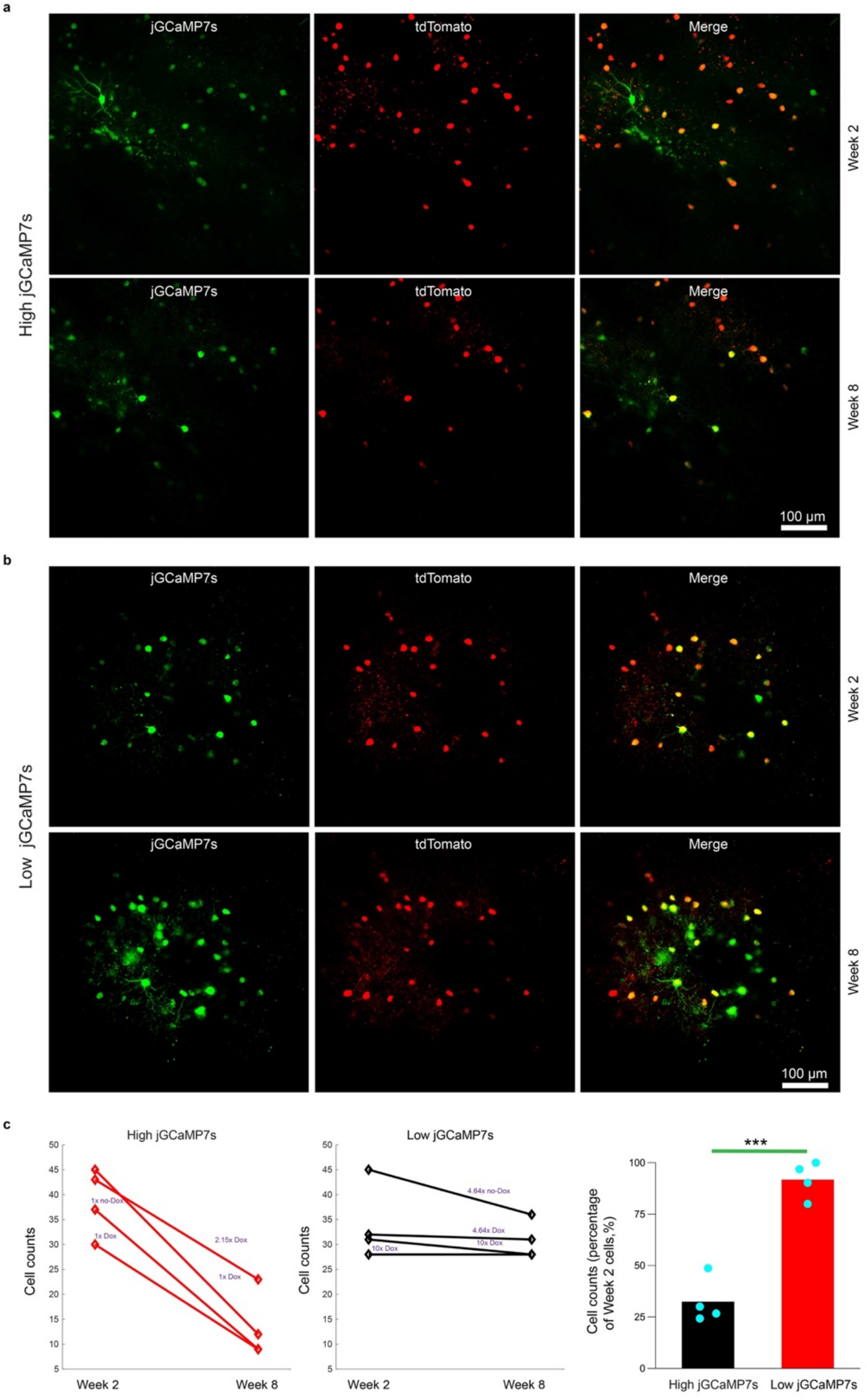
a
High jGCaMP7s
jGCaMP7s
tdTomato
Merge
Week 2
jGCaMP7s
tdTomato
Merge
Week 8
100 μm
b
Low jGCaMP7s
jGCaMP7s
tdTomato
Merge
Week 2
jGCaMP7s
tdTomato
Merge
Week 8
100 μm
c
High jGCaMP7s
Cell counts
50
45
40
35
30
25
20
15
10
5
1x no-Dox
1x Dox
2.15x Dox
1x Dox
Week 2
Week 8
Low jGCaMP7s
Cell counts
50
45
40
35
30
25
20
15
10
5
4.64x no-Dox
4.64x Dox
10x Dox
10x Dox
Week 2
Week 8
***
Cell counts (percentage of Week 2 cells,%)
100
75
50
25
0
High jGCaMP7s
Low jGCaMP7s

Supplementary Fig. 3: Titration of jGCaMP7s AAV concentration to minimize toxicity from jGCaMP7s overexpression. a, Representative two-photon images of neurons expressing tdTomato and jGCaMP7s, with the Flp-dependent AAV1 expressing jGCaMP7s injected at a higher concentration (2.15x diluted from maximum), with doxycycline administered. Top row: 2 weeks after injection. Bottom row: the same FOV at 8 weeks after injection. Numerous cells visible at 2 weeks are no longer seen at 8 weeks. Scale bar: 100 um. **b,** Representative two-photon images from another mouse, in which the Flp-dependent AAV1 expressing jGCaMP7s had been injected at a lower concentration (4.64x diluted from maximum), with doxycycline administered. Top row: 2 weeks after injection. Bottom row: the same FOV at 8 weeks after injection. Almost all cells seen at 2 weeks are still present at 8 weeks. Scale bar: 100 µm. Images from **a-b** are representative of four independent experiments that yielded similar results. **c,** Counts of jGCaMP7s- and tdTomato-expressing neurons infected with high-concentration (left graph) and low-concentration (middle graph) jGCaMP7s AAV. Each data point was obtained from one FOV from each animal (n=4 independent experiments). Virus dilution factor and doxycycline condition are marked on the top of each line; for example, '10x, Dox' means the mouse received the 10-fold dilution of jGCaMP7s AAV and doxycycline food two weeks after the second injection. The surviving cell percentage is shown in the right graph; there is a significant decrease in the high concentration of jGCaMP7s group (***$p=0.00036<0.001$, one-way ANOVA test).

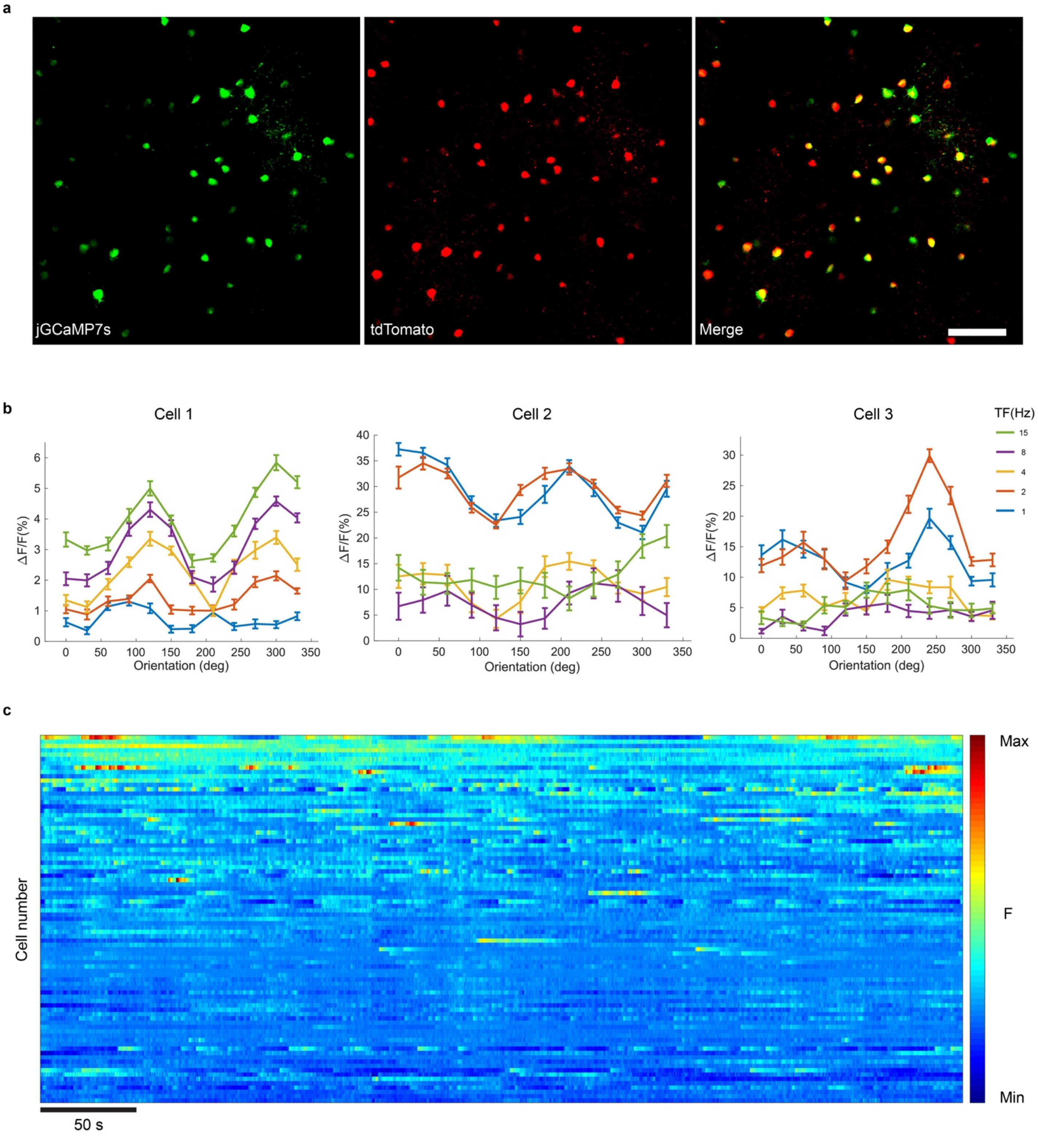

Supplementary Fig. 4: Additional examples of two-photon functional imaging of labeled cells in mouse visual cortex. a, Representative two-photon images of neurons expressing jGCaMP7s and tdTomato at 3 weeks. Scale bar: 100 µm. Images are representative of two independent experiments that yielded similar results. **b,** Direction tuning curves of three different jGCaMP7s-expressing neurons obtained with drifting gratings presented at 12 directions of motion and 5 temporal frequencies (TF), repeated 10 times (mean ΔF/F ± s.e.m.) at 8 weeks; color coding and axis labels are as in Fig. 6. **c,** Single-cell fluorescence time courses for 85 cells over the first 480 s of visual stimulation, showing robust spontaneous and evoked activity. Scale bar: 50 s.

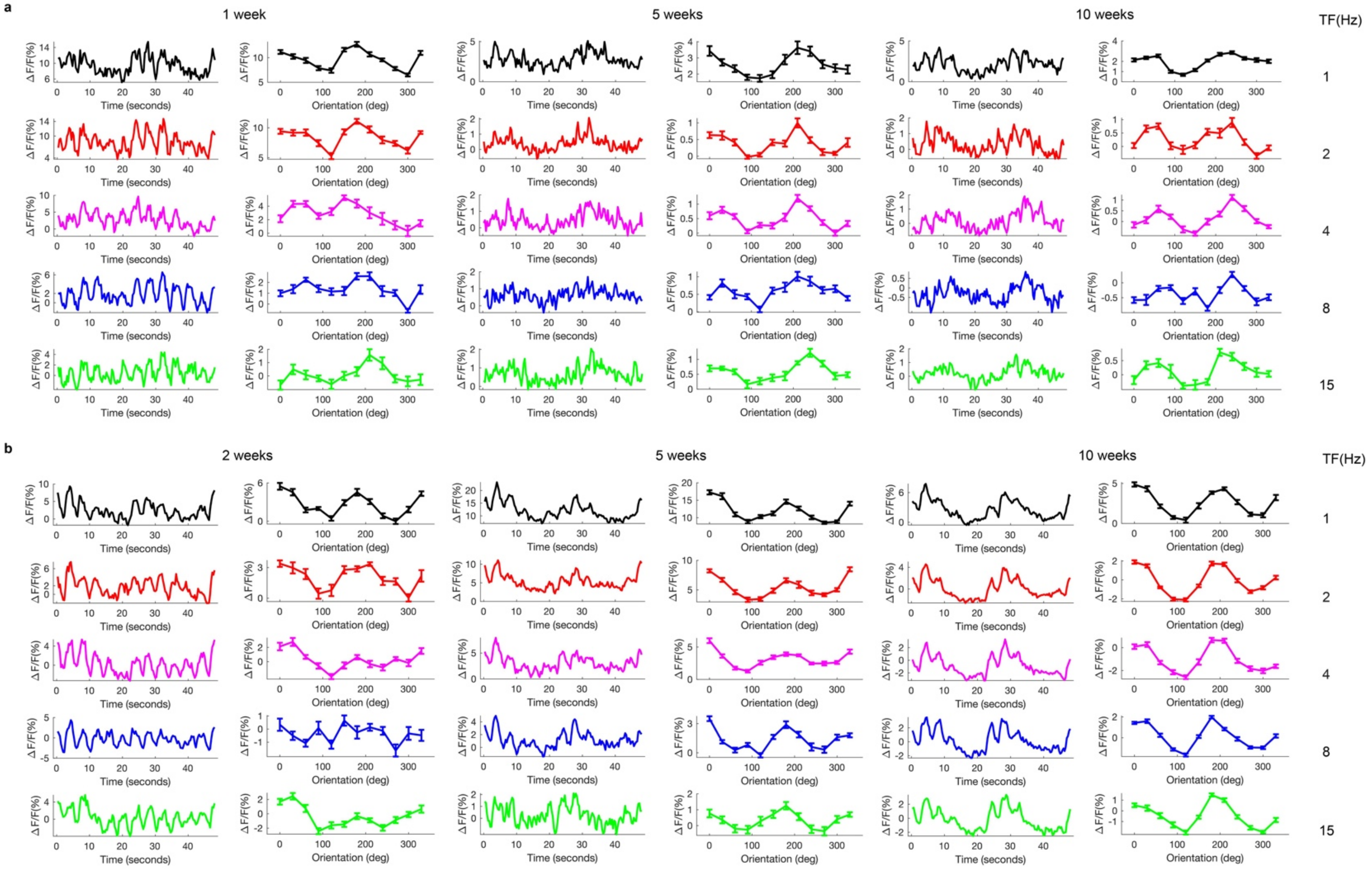

Supplementary Fig. 5: jGCaMP7s signals and tuning curves of two example V1 neurons over multiple imaging sessions. a, jGCaMP7s signals and tuning curves of a "week 1" cell (i.e., in which jGCaMP7s and tdTomato fluorescence could be detected seven days after RVΔGL-Flpo(EnvA) and AAV1-syn-F14F15S-jGCaMP7s injection). These data were obtained with drifting gratings presented at 12 directions of motion and 5 temporal frequencies (TF), repeated 10 times (tuning curve: mean ΔF/F ± s.e.m; jGCaMP7s signals: mean ΔF/F) in three different imaging sessions (left: week 1; middle: week 5; right: week 10). **b,** jGCaMP7s signals and tuning curves of a "week 2" cell (the same cell as used for Fig. 6d). These data were obtained with drifting gratings presented at 12 directions of motion and 5 temporal frequencies (TF), repeated 10 times (tuning curve: mean ΔF/F ± s.e.m; jGCaMP7s signals: mean ΔF/F) in three different imaging sessions (left: week 2; middle: week 5; right: week 10).

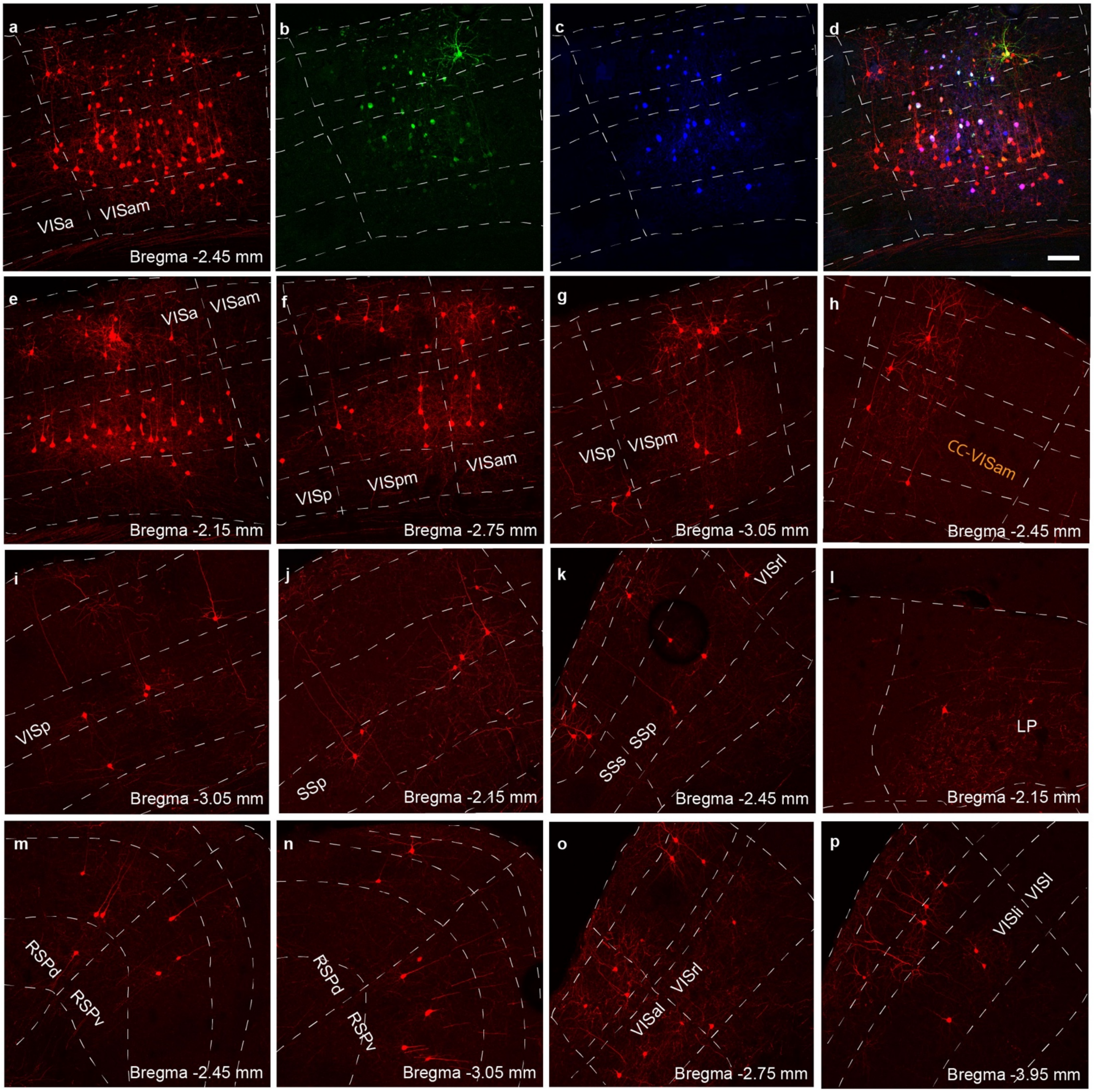

Supplementary Fig. 6: Example confocal images of injection site and input regions in mouse used for longitudinal functional calcium imaging. See Fig. 6a for diagram of experimental design. These images show that inclusion of the jGCaMP7s AAV did not prevent successful monosynaptic tracing. **a-d,** Injection site in V1. **a,** tdTomato, **b),** jCaMP7s, **c,** mTagBFP2, **d,** merge. Scale bar: 100 µm, applies to all panels. **e-p,** Inputs to parvalbumin-expressing V1 neurons are found in many different brain regions: other visual areas (**e-g**, **i**, **o-p**), contralateral cortex (**h**), somatosensory areas (**j-k**), thalamus (**l**), and retrosplenial areas (**m-n**). VISa, anterior visual area; VISam, anteromedial visual area; VISp: Primary visual area; VISpm: Posteromedial visual area; CC-VISam: Contralateral cortex -Anteromedial visual area; SSs: Supplemental somatosensory area; SSp: Primary somatosensory area; LP: Lateral posterior nucleus of the thalamus; RSPd: Retrosplenial area, dorsal part; RSPv: Retrosplenial area, ventral part; VISal: Anterolateral visual area; VISrl: Rostrolateral visual area; VISli: Laterointermediate visual area; VISl: Lateral visual area. Images are representative of two independent experiments that yielded similar results.

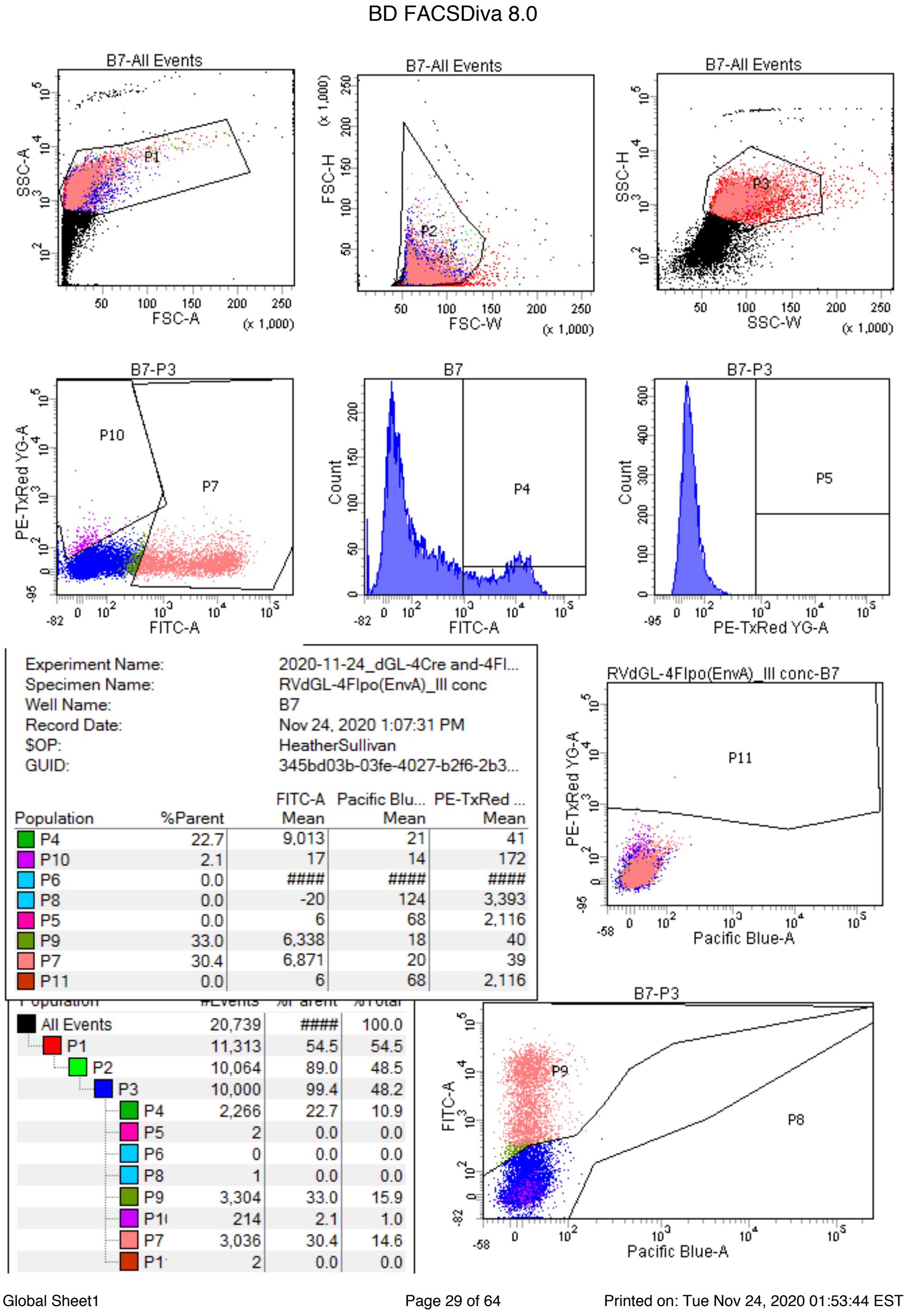

Experiment Name:	2020-11-24_dGL-4Cre and-4Fl...
Specimen Name:	RVdGL-4Flpo(EnvA)_III conc
Well Name:	B7
Record Date:	Nov 24, 2020 1:07:31 PM
$OP:	HeatherSullivan
GUID:	345bd03b-03fe-4027-b2f6-2b3...

Population	%Parent	FITC-A Mean	Pacific Blu... Mean	PE-TxRed ... Mean
P4	22.7	9,013	21	41
P10	2.1	17	14	172
P6	0.0	####	####	####
P8	0.0	-20	124	3,393
P5	0.0	6	68	2,116
P9	33.0	6,338	18	40
P7	30.4	6,871	20	39
P11	0.0	6	68	2,116

Population	#Events	%Parent	%Total
All Events	20,739	####	100.0
P1	11,313	54.5	54.5
P2	10,064	89.0	48.5
P3	10,000	99.4	48.2
P4	2,266	22.7	10.9
P5	2	0.0	0.0
P6	0	0.0	0.0
P8	1	0.0	0.0
P9	3,304	33.0	15.9
P1[illegible]	214	2.1	1.0
P7	3,036	30.4	14.6
P1[illegible]	2	0.0	0.0

Supplementary Fig. 7: Representative example of FACS data for titering viruses. This example, included as stipulated by the Nature Portfolio Reporting Summary, shows FACS analysis data for one dilution of RVΔGL-Flpo(EnvA), used to infect HEK 293T cells which were immunostained for the rabies virus nucleoprotein using a blend of FITC-conjugated monoclonal antibodies (see Methods). The middle histogram in the second row shows the characteristic bimodal distribution (less distinct with second-generation RV vectors than with first-generation ones) with the uninfected cells in the mode on the left and infected ones in the one on the right. Gates are set by comparison with negative control wells of uninfected cells.
