# Supplementary material for: Long-term labeling and imaging of synaptically connected neuronal networks in vivo using double-deletion-mutant rabies viruses
Source: Nat Neurosci. 2024 Jan 11;27(2):373–83. doi: 10.1038/s41593-023-01545-8 (PMC10849964; doi:10.1038/s41593-023-01545-8)
Supplement: Supplementary file 2 — Reporting Summary [file 41593_2023_1545_MOESM2_ESM.pdf]

Corresponding author(s): Ian Wickersham
Last updated by author(s): Nov 26, 2023

Reporting Summary

Nature Portfolio wishes to improve the reproducibility of the work that we publish. This form provides structure for consistency and transparency in reporting. For further information on Nature Portfolio policies, see our Editorial Policies and the Editorial Policy Checklist.

Statistics

For all statistical analyses, confirm that the following items are present in the figure legend, table legend, main text, or Methods section.

n/a	Confirmed	
☐	☒	The exact sample size (*n*) for each experimental group/condition, given as a discrete number and unit of measurement
☐	☒	A statement on whether measurements were taken from distinct samples or whether the same sample was measured repeatedly
☐	☒	The statistical test(s) used AND whether they are one- or two-sided<br>*Only common tests should be described solely by name; describe more complex techniques in the Methods section.*
☐	☒	A description of all covariates tested
☐	☒	A description of any assumptions or corrections, such as tests of normality and adjustment for multiple comparisons
☐	☒	A full description of the statistical parameters including central tendency (e.g. means) or other basic estimates (e.g. regression coefficient) AND variation (e.g. standard deviation) or associated estimates of uncertainty (e.g. confidence intervals)
☐	☒	For null hypothesis testing, the test statistic (e.g. F, t, r) with confidence intervals, effect sizes, degrees of freedom and P value noted<br>*Give P values as exact values whenever suitable.*
☒	☐	For Bayesian analysis, information on the choice of priors and Markov chain Monte Carlo settings
☒	☐	For hierarchical and complex designs, identification of the appropriate level for tests and full reporting of outcomes
☒	☐	Estimates of effect sizes (e.g. Cohen's *d*, Pearson's *r*), indicating how they were calculated

Our web collection on statistics for biologists contains articles on many of the points above.

Software and code

Policy information about availability of computer code

Data collection	For longitudinal two-photon imaging of live monosynaptic tracing, injection sites were imaged on a Prairie/Bruker Ultima IV In Vivo two-photon microscope controlled by Prairie View 5.4 software. Visual stimuli were generated in Matlab (R2015R version) with custom software based on Psychtoolbox 3.0.17 (http://psychtoolbox.org). All confocal images were acquired with a confocal microscope (Zeiss, LSM 900). The images for cell counts were acquired with an epifluorescence microscope (Zeiss Imager.Z2). Slice electrophysiology data were collected using pClamp 10 software (Molecular Devices). Flow cytometry data were collected using BD FACSDiva v.9.0 software.
Data analysis	For the example images of labeled cells, maximum intensity projections (stacks of 150-400 µm) were made with Fiji software. Cell counting was performed with the ImageJ Cell Counter plugin. Plots of cell counts were made with Origin 7.0 software (OriginLab, Northampton, MA) or Prism 9 (GraphPad Software, San Diego, California). The statistical analysis was done using Microsoft Excel for Mac version 16.42. Confocal images were analyzed using analyzed using ImageJ 2.0.0. Slice electrophysiology data were analyzed using Clampfit 10 software (Molecular Devices).

For manuscripts utilizing custom algorithms or software that are central to the research but not yet described in published literature, software must be made available to editors and reviewers. We strongly encourage code deposition in a community repository (e.g. GitHub). See the Nature Portfolio guidelines for submitting code & software for further information.

Data

Policy information about availability of data

All manuscripts must include a data availability statement. This statement should provide the following information, where applicable:

- Accession codes, unique identifiers, or web links for publicly available datasets
- A description of any restrictions on data availability
- For clinical datasets or third party data, please ensure that the statement adheres to our policy

All cell counts and statistical analyses are provided in Supplementary Information. The new plasmids described in this paper have been deposited with Addgene with the accession numbers given in Methods. The TRE-CB mouse line is available from the Jackson Laboratory (accession number 036974).

Field-specific reporting

Please select the one below that is the best fit for your research. If you are not sure, read the appropriate sections before making your selection.

☒ Life sciences ☐ Behavioural & social sciences ☐ Ecological, evolutionary & environmental sciences

For a reference copy of the document with all sections, see nature.com/documents/nr-reporting-summary-flat.pdf

Life sciences study design

All studies must disclose on these points even when the disclosure is negative.

Sample size	Sample sizes for each type of experiment in this study are consistent with previously published work (e.g., PMID 37989085) and were the maximum practical sizes given the large number of conditions tested and the constraintsof limited resources.
Data exclusions	Animal exclusions only affected the longitudinal experiments in this work, and the criteria were animal health/death. For two-photon structural imaging experiments, one mouse in the no-dox group was excluded because of little label, ascribed to misaligned injections of helper AAVs and RV.
Replication	The number of times each experiment was repeated is indicated in the text and legend, and all experimental findings were reliably reproduced.
Randomization	Mice were pseudorandomly assigned to each condition, with approximate balancing of sexes and ages across conditions, subject to the availability of transgenic mice of the correct genotypes.
Blinding	No blinding was used, as the lab members preparing the tissue were the same ones doing the quantification.

Reporting for specific materials, systems and methods

We require information from authors about some types of materials, experimental systems and methods used in many studies. Here, indicate whether each material, system or method listed is relevant to your study. If you are not sure if a list item applies to your research, read the appropriate section before selecting a response.

Materials & experimental systems

n/a	Involved in the study	
☐	☒	Antibodies
☐	☒	Eukaryotic cell lines
☒	☐	Palaeontology and archaeology
☐	☒	Animals and other organisms
☒	☐	Human research participants
☒	☐	Clinical data
☒	☐	Dual use research of concern

Methods

n/a	Involved in the study	
☒	☐	ChIP-seq
☐	☒	Flow cytometry
☒	☐	MRI-based neuroimaging

Antibodies

Antibodies used	Chicken anti-GFP (Aves Labs GFP-1020) 1:500, guinea pig anti-parvalbumin (Synaptic Systems 195004) 1:1000, sheep anti-tyrosine hydroxylase (Millipore AB1542)) 1:1000, with secondary antibodies donkey anti-chicken Alexa Fluor 488 (Jackson Immuno 703-545-155) 1:200, donkey anti-guinea pig, AlexaFluor 647 conjugated (Jackson Immuno 706-605-148) 1:200, and donkey anti-sheep, AlexaFluor 647 conjugated (Jackson Immuno 713-605-147)) 1:200.
Validation	All antibodies are validated in our previous publication {Lavin et al., Front Synaptic Neurosci 12, 6 (2020)}.

Eukaryotic cell lines

Policy information about cell lines

Cell line source(s)	HEK 293T/17 and BHK-21 cells were obtained from ATCC. The BHK-B19L, BHK-B19L-TVA950, and BHK-EnvA2-TTBL2 cell lines were made in-house and derived from these two lines.
Authentication	Cell lines obtained from ATCC authenticated by ATCC (Post-freeze viability using cell count using Trypan Blue stain method, visual observation of morphology and growth properties, testing for mycoplasma contamination using Hoechst DNA stain (indirect) method, agar culture (direct) method, and PCR-based assay, species determination: COI assay (interspecies), sterility test (BacT/ALERT 3D) in iAST bottle (aerobic) at 32.5°C and iNST bottle (anaerobic) at 32.5°C).<br>Cell lines made in-house were derived from these ATCC-authenticated cell lines and extensively validated by testing their ability to produce, or report activity of, viruses made from sequence-verified plasmids.
Mycoplasma contamination	Cell lines obtained from ATCC were tested for mycoplasma contamination by ATCC. Cell lines were not subsequently tested for mycoplasma contamination.
Commonly misidentified lines<br>(See ICLAC register)	No commonly misidentified cell lines were used.

Animals and other organisms

Policy information about studies involving animals; ARRIVE guidelines recommended for reporting animal research

Laboratory animals	PV-Cre, DAT-IRES-Cre, DAT-P2A-Flpo, and Ai14 were purchased from Jackson Laboratory (catalog #s 017320, 006660, 035436, and 007914). C57BL/6J was purchased from Jackson Laboratory (catalog # 000664); Ai65F (Jackson Laboratory with catalog # 032864); the L-expressing mouse line TRE-CB (TRE-tight-mCardinal-P2A-B19L) was generated by the Mouse ES Cell & Transgenic Facility at the Koch Institute for Integrative Cancer Research at MIT using ES cells genetically modified by the authors.<br>Strains, sexes, and ages (at time of injection) of mice used by experiment were as follows (all in C57BL/6J background):<br>Corticostriatal tracing (Figure 1):<br>Ai14 het: 10F, 2M, age range 6-11 weeks<br>Ai65F het: 7F, 1M, age range 14-28 weeks<br>Ai65F homo: 2F, 2M, age 17 weeks<br>Ai14 x TRE-CB het/het: 18F, 6M, age range 8-13 weeks<br>Ai65F x TRE-CB het/het: 13F, 11M, age range 6-26 weeks<br>Tracing in Cre mice (Figure 2):<br>Ai65F x TRE-CB het/het: 5F, 11M, age range 7-17 weeks<br>DAT-Cre x Ai65F het/het: 4F, 4M, age range 8-11 weeks<br>DAT-Cre x Ai65F x TRE-CB het/het/het: 13F, 15M, age range 5-9 weeks<br>PV-Cre x Ai65F het/het: 4 F, 4M, age range 6-7 weeks<br>PV-Cre x Ai65F x TRE-CB het/het/het: 19F, 9M, age range 6-22 weeks<br>Tracing in Flpo mice (Figure 3):<br>Ai65F x TRE-CB het/het: 3F, 1M, age 7 weeks<br>DAT-Flpo x Ai14 het/het: 2F, 2M, age range 7-19 weeks<br>DAT-Flpo x Ai14 x TRE-CB het/het: 3F, 9M, age range 6-13 weeks<br>Structural two-photon imaging (Figure 4):<br>PV-Cre x Ai65F x TRE-CB het/het/het: 6M, age range 15-20 weeks<br>Whole-cell electrophysiology (Figure 5):<br>PV-Cre x Ai65F het/het: 8F, 1M, age range 28-47 weeks<br>PV-Cre x Ai65F x TRE-CB het/het/het: 3F, 1M, age 6 weeks<br>Functional two-photon imaging (Figure 6):<br>PV-Cre x Ai65F x TRE-CB het/het/het: 2M, age range 10-14 weeks<br>Two-photon imaging of RVΔG-Flpo & RVΔGL-Flpo (Supplementary Fig. 1):<br>Ai65F het: 3F, 4M, age range 7-29 weeks
Wild animals	No wild animals were used in the study.
Field-collected samples	No field-collected samples were used in the study.
Ethics oversight	All experiments involving animals were conducted according to NIH guidelines and approved by the MIT Committee for Animal Care.

Note that full information on the approval of the study protocol must also be provided in the manuscript.

Flow Cytometry

Plots

Confirm that:

☒ The axis labels state the marker and fluorochrome used (e.g. CD4-FITC).

☒ The axis scales are clearly visible. Include numbers along axes only for bottom left plot of group (a 'group' is an analysis of identical markers).

☒ All plots are contour plots with outliers or pseudocolor plots.

☒ A numerical value for number of cells or percentage (with statistics) is provided.

Methodology

Sample preparation	For titering, HEK 293T cells were fixed in 1% PFA, resuspended, and analyzed on a BD LSR II. For sorting, the cells were expanded into two or three 15c plates, then resuspended and sorted on a BD FACS Aria.
Instrument	BD LSR II, BD FACS Aria
Software	BD FACSDiva v.9.0
Cell population abundance	Collected the brightest 2% or 5% of fluorescent protein-expressing cells.
Gating strategy	See Supplementary Fig. S7. Gates are set by comparison with negative control wells of uninfected cells, with with the uninfected cells in the mode on the left and infected ones in the one on the right in the selected region.

☒ Tick this box to confirm that a figure exemplifying the gating strategy is provided in the Supplementary Information.
